# The organ-specific differential roles of rice DXS and DXR, the first two enzymes of the MEP pathway, in carotenoid metabolism in *Oryza sativa* leaves and seeds

**DOI:** 10.1186/s12870-020-02357-9

**Published:** 2020-04-15

**Authors:** MK You, YJ Lee, JK Kim, SA Baek, YA Jeon, SH Lim, SH Ha

**Affiliations:** 1 Department of Genetic Engineering and Graduate School of Biotechnology, College of Life Sciences, Kyung Hee University, Yongin, 17104 Republic of Korea; 2 Division of Life Sciences and Bio-Resource and Environmental Center, Incheon National University, Incheon, 22012 Republic of Korea; 3 College of Agriculture and Life Sciences, Chungnam National University, Daejeon, 34134 Republic of Korea; 4 National Academy of Agricultural Science, Rural Development Administration, Jeonju, 54874 Republic of Korea

**Keywords:** *OsDXS*, *OsDXR*, A rate-limiting step, MEP pathway, Carotenoids, Rice

## Abstract

**Background:**

Deoxyxylulose 5-phosphate synthase (DXS) and deoxyxylulose 5-phosphate reductoisomerase (DXR) are the enzymes that catalyze the first two enzyme steps of the methylerythritol 4-phosphate (MEP) pathway to supply the isoprene building-blocks of carotenoids. Plant DXR and DXS enzymes have been reported to function differently depending on the plant species. In this study, the differential roles of rice *DXS* and *DXR* genes in carotenoid metabolism were investigated.

**Results:**

The accumulation of carotenoids in rice seeds co-expressing *OsDXS2* and *stPAC* was largely enhanced by 3.4-fold relative to the *stPAC* seeds and 315.3-fold relative to non-transgenic (NT) seeds, while the overexpression of each *OsDXS2* or *OsDXR* caused no positive effect on the accumulation of either carotenoids or chlorophylls in leaves and seeds, suggesting that OsDXS2 functions as a rate-limiting enzyme supplying IPP/DMAPPs to seed carotenoid metabolism, but OsDXR doesn’t in either leaves or seeds. The expressions of *OsDXS1*, *OsPSY1*, *OsPSY2*, and *OsBCH2* genes were upregulated regardless of the reductions of chlorophylls and carotenoids in leaves; however, there was no significant change in the expression of most carotenogenic genes, even though there was a 315.3-fold increase in the amount of carotenoid in rice seeds. These non-proportional expression patterns in leaves and seeds suggest that those metabolic changes of carotenoids were associated with overexpression of the *OsDXS2*, *OsDXR* and *stPAC* transgenes, and the capacities of the intermediate biosynthetic enzymes might be much more important for those metabolic alterations than the transcript levels of intermediate biosynthetic genes are. Taken together, we propose a ‘Three Faucets and Cisterns Model’ about the relationship among the rate-limiting enzymes OsDXSs, OsPSYs, and OsBCHs as a “Faucet”, the biosynthetic capacity of intermediate metabolites as a “Cistern”, and the carotenoid accumulations as the content of “Cistern”.

**Conclusion:**

Our study suggests that OsDXS2 plays an important role as a rate-limiting enzyme supplying IPP/DMAPPs to the seed-carotenoid accumulation, and rice seed carotenoid metabolism could be largely enhanced without any significant transcriptional alteration of carotenogenic genes. Finally, the “Three Faucets and Cisterns model” presents the extenuating circumstance to elucidate rice seed carotenoid metabolism.

## Background

Plant terpenoids are the most diverse group of secondary metabolites and participate in essential functions, such as photosynthesis (carotenoids, chlorophylls, and prenylquinones), regulation of growth and development (cytokinin, gibberellins, abscisic acid, and strigolactones), respiration (ubiquinone), and secondary roles responding to environmental conditions [1, 2]. Also, a large number of terpenoids have been widely used in the industrial or medicinal fields as flavors, pigments, polymers, or drugs [3]. Terpenoids are generated by the sequential condensation and modification of two building-blocks, isopentenyl pyrophosphate (IPP, C_5_) and dimethylallyl pyrophosphate (DMAPP, C_5_), via the methylerythritol 4-phosphate (MEP) pathway in plastids and the mevalonic acid (MVA) pathway in cytosols [3]. In the first step, the pyruvate (C_3_) and glyceraldehyde 3-phosphate (GAP, C_3_) derived from glycolysis are condensed into deoxyxylulose 5-phosphate (DXP, C_5_) by a DXP synthase (DXS), and DXPs are subsequently converted into MEP (C_5_) by a DXP reductoisomerase (DXR) [4].

Plant *DXS* and *DXR* genes have been reported to function in terpenoid metabolism in a species- or organ-specific manner. In Arabidopsis, both AtDXS and AtDXR function as rate-limiting enzymes to enhance the leaf carotenoid and chlorophyll contents [4, 5], but their functions vary in a plant species-specific manner. For example, the heterologous expression of either *AtDXS* or *AtDXR* increased the amount of abietane diterpenes (C_20_) in *Salvia sclarea* hairy roots [6], but only AtDXS caused an increase in carotenoids and chlorophylls in leaves and roots of *Daucus carota* [7], and AtDXS in *Lavandula latifolia* significantly increased the monoterpene content (C_10_) but did not affect the amount of carotenoids and chlorophylls [8]. In addition, AtDXR affect neither the carotenoid, chlorophyll, nor monoterpene content in leaves of *L. latifolia* [9] or carrot [7]. On the other hand, overexpression of either the *Amomum villosum* Lour DXR gene (*AvDXR*) or the cyanobacteria (*Synechosystis* sp. strain PCC6803) DXR gene (*SyDXR*) enhanced the accumulation of carotenoid and chlorophyll in tobacco (*Nicotiana tabacum*) leaves. Similarly, the two tobacco genes *NtDXR1* and *NtDXR2* have also been reported to enhance the carotenoid and chlorophyll contents in tobacco leaves [10–12]. In this way, the differential roles of DXS and DXR have been elucidated in several plant systems, but the roles of DXS and DXR genes have largely remained unknown in rice plants.

The gene family of DXS enzymes has been classified into three groups. The type I-DXS group plays essential roles in the biosynthesis of housekeeping and photosynthetic terpenoids such as chlorophylls and carotenoids in leaves of *Arabidopsis*, Medicago (*Medicago truncatula*), rice and maize (*Zea mays*), and their gene expressions are dependent on the light condition [13–16]. The type II-DXS group plays secondary and ecological roles in the production of functional terpene metabolites, such as ginkgolide in ginkgo (*Ginkgo biloba*), oleoresin in Norway spruce (*Picea abies*), phytoalexin in rice, apocarotenoids in mycorrhizal roots of Medicago, and carotenoids in yellow kernels of maize [13, 17–20]. Type III-DXS enzymes have tentative roles specifically in the *Poaceae* family and a few dicots among angiosperms [13, 15, 21, 22]. The DXR enzyme is encoded by a single gene in most plants, except tobacco (*N. tabacum*), *Hevea brasiliensis*, and soybean (*Glycine max*), and it plays essential roles in the development and survival of plants [23–25].

Carotenoids, a large family of tetraterpenoids (C_40_), are abundant in photosynthetic and non-photosynthetic plant tissues [26], and are also essential nutrients for humans as they are the precursors of pro-vitamin A [27]. Since carotenoids are enriched in photosynthetic green parts but lacking in non-photosynthetic seed endosperms in rice plants [28, 29], several “Golden Rice” varieties have been genetically engineered in which carotenoids, such as β-carotene, are enriched in endosperms [29–32] to enhance the nutritional value of rice as a staple cereal. In maize plants (*Zea mays* L.), the expression levels of the three maize *DXS* genes were examined in different organs, including the mature kernels of two varieties with white and yellow grain [13]. The highest level of *ZmDXS2* expression was observed in yellow kernels compared with its expression level in white kernels, whereas *ZmDXS1* was not expressed, and *ZmDXS3* was expressed in mature kernels of both varieties, suggesting that *ZmDXS2* might be important for the carotenoid enrichment in yellow kernels. To increase the accumulation efficiency of carotenoids in rice seed endosperms, the MEP pathway supplying the major building-blocks, IPP/DMAPP, to the carotenoid pathway has been issued, and the heterologous expression of *AtDXS* has shown the possibility that the increase in DXS enzyme could derive the enhancement of carotenoid accumulation in rice endosperms [33]. However, even though the “Golden rice” varieties have been developed for years, there has been no report of the rate-limiting enzymes of the rice MEP pathway, which function to increase the flux of the isoprene building-blocks IPP/DMAPP into carotenoid metabolism.

In this study, we investigated the differential roles of rice DXS and DXR, the first two enzymes of the MEP pathway, and determined their influences on rice carotenoid metabolism in leaves and seeds. Considering the genome editing era, our studies provide useful information on gene candidates for genome editing.

## Results

### Molecular characterization of rice genes encoding DXS and DXR

**Fig. 1:**
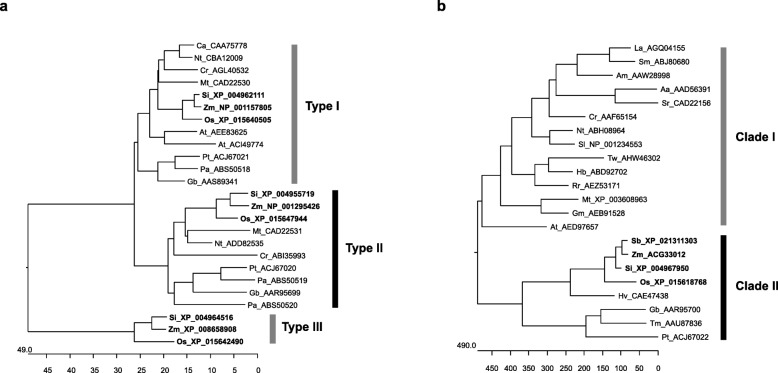
Phylogenetic relationships of plant **a** deoxyxylulose 5-phosphate synthases (DXSs) and **b** deoxyxylulose 5-phosphate reductoisomerases (DXRs). The accession numbers of plant DXSs and DXRs are displayed with the abbreviated names of plant species as follows: Aa, *Artemisia annua*; Am, *Amomum villosum* Lour; At, *Arabidopsis thaliana*; Ca, *Capsicum annuum*; Cr, *Catharanthus roseus*; Do, *Dendrobium officinal*; Gb, *Ginkgo biloba*; Gm, *Glycine max*; Hb, *Hevea brasiliensis*; Hv, *Hordeum vulgare*; La, *Lavandula angustifolia*; Mt, *Medicago truncatula*; Nt, *Nicotiana tabacum*; Os, *Oryza sativa*; Pa, *Picea abies*; Pt, *Pinus taeda*; Rr, *Rosa rugosa*; Sb, *Sorghum bicolor*; Si, *Setaria italica*; Sl, *Solanum lycopersicum*; Sm, *Salvia militorrhiza*; Sr, *Stevia rebaudiana*; Tm, *Taxus x media*; Tw, *Tripterygium wilfordii*; Zm, *Zea mays*. Their deduced amino acid sequences were aligned using the ClustalV algorithm of the MegAlign program (DNASTAR, Inc.). Type II proteins, including OsDXS2, and clade II proteins, including OsDXR, are distinguished by black and gray bars, respectively, and proteins belonging to the *Poaceae* family including rice are in bold

In rice, the three genes that encode DXS proteins are Os05g33840 (XP_015640505), Os06g05100 (XP_015642490), and Os07g09190 (XP_015647944). The predicted amino sequences of three rice *DXS* genes were phylogenetically analyzed with plant DXS protein sequences and were independently classified as type I, II, or III, corresponding to OsDXS1 (XP_015640505), OsDXS2 (XP_015642490), and OsDXS3 (XP_015647944), respectively (Fig. 1a). The three rice DXSs are closely related to DXS in foxtail millet (*Setaria italica*) and maize, which belong to the *Poaceae* family. OsDXS2 showed the highest sequence similarity (87.5%) to ZmDXS2 (NP_001295426), therefore it was chosen to study its influence on rice carotenoid metabolism in leaves and seeds. Similar to DXR in other plants, rice DXR is encoded by the single copy gene *OsDXR* (XP_015618768, Os01g01710) and phylogenetically categorized into plant DXR clade II, which includes the tree plants gingko, yew, and pine, and the *Poaceae* family plants sorghum (*Sorghum bicolor*), maize, foxtail millet, and barley (*Hordeum vulgare*). On the other hand, rice DXR is not categorized into clade I, which includes herbage plants (Fig. 1b). The predicted amino acid sequence of OsDXS2 has a conserved core domain for thiamine pyrophosphate (TPP)-binding and two residues of histidine and tyrosine that participate in the active site (Additional file 1: Figure S1). In addition, OsDXR has three highly conserved domains, including a proline-rich domain, two NADPH binding sites, and two substrate-binding sites (Additional file 2: Figure S2).

### Expression profile of *OsDXS*s and *OsDXR* in different tissues

**Fig. 2:**
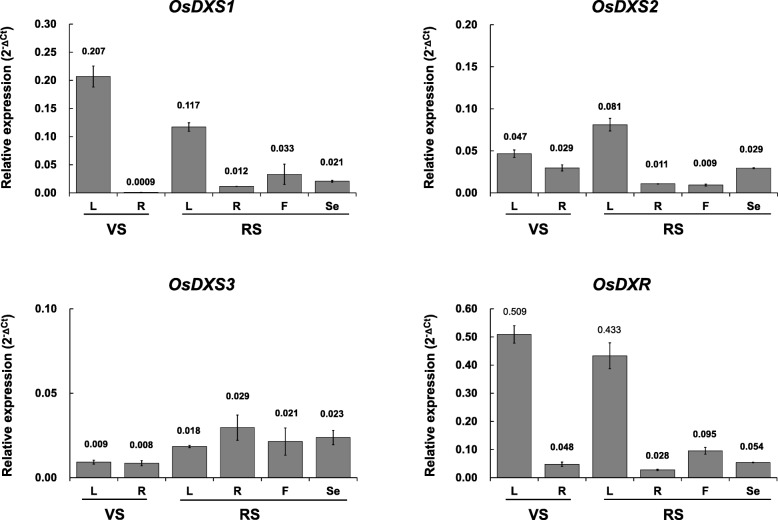
Spatial and temporal expression of rice genes encoding deoxyxylulose 5-phosphate synthase (DXS) or deoxyxylulose 5-phosphate reductoisomerase (DXR). Endogenous transcript levels of the *OsDXS1*, *OsDXS2*, *OsDXS3*, and *OsDXR* genes were examined by qRT-PCR using total RNA isolated from various tissues, including leaves (L), roots (R), and florets (F) of vegetative stage and reproductive stage tissues, and seeds (Se) harvested at 40 days after flowering using gene-specific primers as shown in Additional file 4: Table S8. All transcript levels measured in three technical replicates were calculated by the ΔCt equation against the *OsUbi5* gene. The results are expressed as the mean ± standard error (SE)

The endogenous expression patterns of *OsDXS*s and *OsDXR* genes were analyzed in leaves and roots of the vegetative stage and in leaves, roots, florets, and seeds of the reproductive stage (Fig. 2). The transcript level of *OsDXS1* was significantly higher in the leaves compared with other tissues of both stages, suggesting its relevant roles in photosynthetic tissues. Similarly, the expression of *OsDXS2* was also higher in the leaves compared with other tissues at both stages. In the roots of the vegetative stage, the expression levels of *OsDXS2, OsDXS1,* and *OsDXS3* were 0.029, 0.0009, and 0.008, respectively (Fig. 2), and that the expression level of *OsDXR* was 0.048 in the roots. Considering an *OsDXR* is a single copy-downstream gene of *OsDXS*, *OsDXS2* was supposed to play important roles as a major DXS enzyme in the roots as a non-photosynthetic tissue. In contrast, *OsDXS3* was constitutively expressed at low levels in most tissues, including seeds. As expected as a single copy-downstream gene of *OsDXS*, *OsDXR* was strongly expressed in the leaves of both developmental stages, and exhibited the highest expression among the four genes in all tissues, regardless of the developmental stage (Fig. 2).

### Constitutive overexpression of either *OsDXS2* or *OsDXR* alone or in combination with the bio-fortified trait of β-carotene accumulation in rice endosperm

**Fig. 3:**
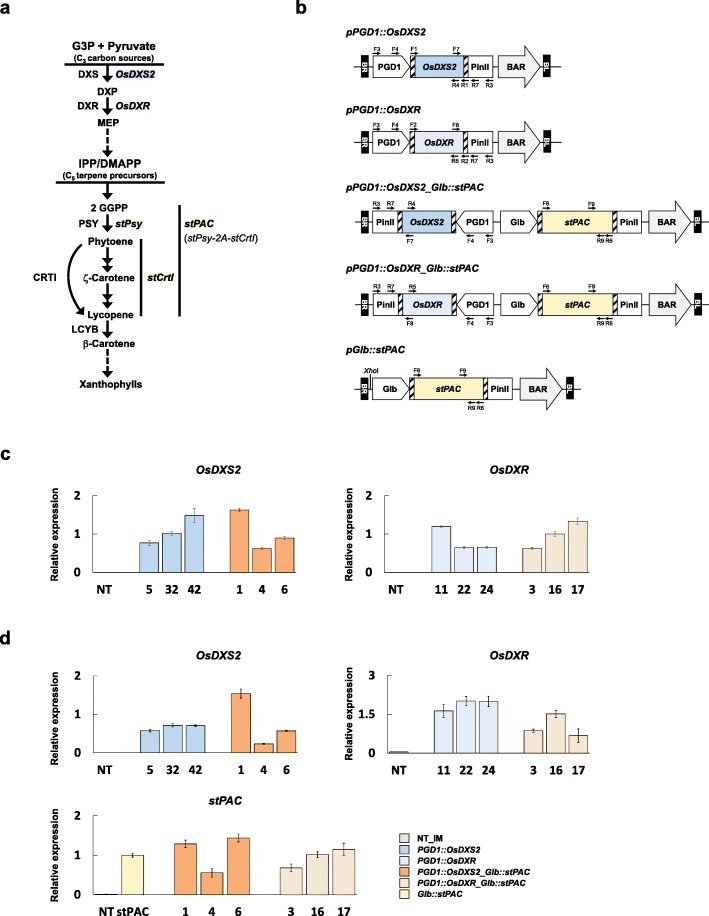
Schematic representation of the carotenoid pathway, the binary vectors used in this study, and transgene expression in leaves and seeds of rice plants. **a** Built-in pathway for carotenoid biosynthesis in rice plants; CRTI, *Pantoea annatis* desaturase; DMAPP, dimethylallyl diphosphate; DXP, deoxyxylulose 5-phosphate; DXR, DXP reductoisomerase; DXS, DXP synthase; IPP, isopentenyl diphosphate; GGPP, geranylgeranyl pyrophosphate; G3P, glyceraldehyde 3-phosphate; LCYB, lycopene β-cyclase; MEP, methylerythritol 4-phosphate; OsDXR, rice DXP reductoisomerase; OsDXS2, rice DXP synthase; PSY, phytoene synthase; *stCrtI*, rice codon-optimized synthetic gene encoding the *Pantoea CrtI* gene; *stPsy*, rice codon-optimized synthetic gene of the *Capsicum* gene encoding PSY; *stPAC*, a recombinant gene of *stPsy* and *stCrt* linked with *2A*, which is the rice codon-optimized foot-and-mouth disease virus 2A peptide. **b** Diagrams of the four vectors for rice transformation of *OsDXS2* and *OsDXR* with the *pGlb::stPAC* vector that was previously used to generate a carotenoid-intensifying trait in rice endosperm [32]. Bacterial attachment *attB* sites needed for Gateway cloning are marked with hatched boxes. BR, right border; BL, left border; PGD1, rice phosphogluconate dehydrogenase promoter; *OsDXS2*, rice DXP synthase 2 gene; *OsDXR*, rice DXP reductoisomerase gene; PinII, 3′ region of the potato proteinase inhibitor II gene; BAR, Bialaphos-resistant gene cassette; Glb, rice globulin promoter; *stPAC*, a recombinant gene of *stPsy-2A-stCrt*. The primer locations used in vector construction, PCR, and qRT-PCR for the transgene analyses in Additional file 3: Figure S3a, Fig. 3c, and Fig. 3d are indicated as arrows, and their information is listed in Additional file 4: Table S7. **c** Transgene expression levels of *OsDXS2* and *OsDXR* were examined by qRT-PCR using total RNA isolated from 10-day-old leaves. **d** Transgene expression levels of *OsDXS2*, *OsDXR*, and *stPAC* were examined by qRT-PCR using total RNA purified from unpolished mature seeds 40 DAF. All results using gene-specific primer pairs F7/R7 for *OsDXS2*, F8/F7 for *OsDXR,* and F9/R9 for *stPAC* were calculated as the mean of three replicates and normalized to the expression of the *OsUbi5* gene, which was amplified using a U5F/U5R primer pair. The primers are indicated in Fig. 3b and listed in Additional file 4: Table S7. NT is a non-transgenic wild type of *Oryza sativa* L. cv. Ilmi, different varieties of transgenic plants are represented in different colors, such as blue, light-blue, orange, light-orange and yellow in the bar graph, and the X-axis labeling consists of three independent-transgenic plant lines

To investigate the differential roles of *OsDXS2* and *OsDXR* in rice carotenoid metabolism in leaves and seeds (Fig. 3a), *PGD1::OsDXS2* and *PGD1::OsDXR* vectors were generated to constitutively overexpress *OsDXS2* (Os07g09190) and *OsDXR* (Os01g01710) (Fig. 3b). Their expression cassettes, either *PGD1::OsDXS2::PinII* or *PGD1::OsDXR::PinII*, were cloned into the *pGlb::stPAC* vector for co-expression with a *stPAC* recombinant gene producing β-carotene in rice endosperms to generate *PGD1::OsDXS2_Glb::stPAC* or *PGD1::OsDXR*_*Glb::stPAC* vectors, respectively (Fig. 3b). Finally, three representative lines for each of the four constructs were selected by considering the integration of the transgene and its copy numbers in leaves of T3 plants (Additional file 3: Figure S3).

All transgene expressions were examined in leaves and seeds of the T4 generation (Fig. 3c and d). The transcript level of *OsDXS2* or *OsDXR* in both organs relative to the NT rice plant demonstrated their constitutive overexpression, and the *stPAC* expression in seeds of *PGD1::OsDXS2_Glb::stPAC* and *PGD1::OsDXR*_*Glb::stPAC* lines verified the integration of the β-carotene-producing pathway (Fig. 3d).

### No positive effect of OsDXS2 and OsDXR on carotenoid- and chlorophyll metabolism in rice leaves

**Fig. 4:**
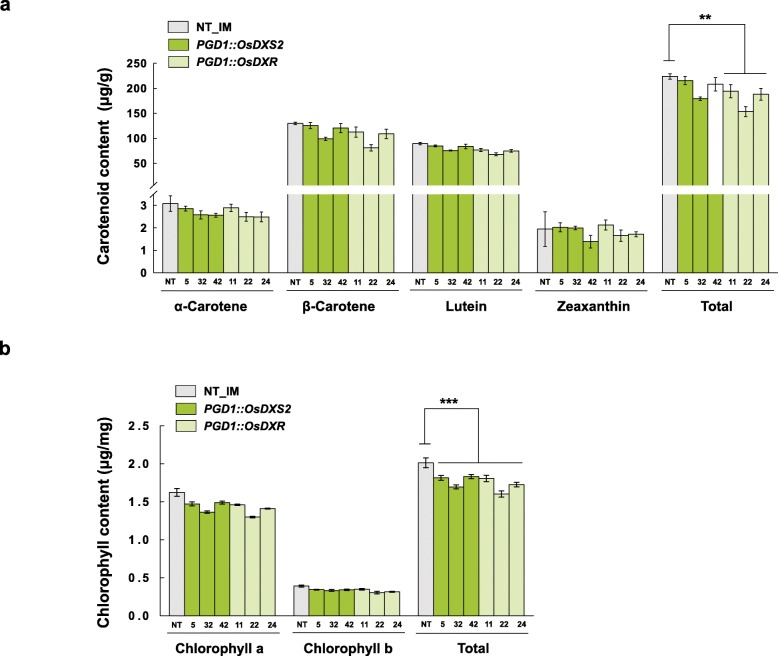
Contents and composition of carotenoids and chlorophylls in the leaves of *PGD1::OsDXS2* and *PGD1::OsDXR* transgenic plants relative to NT plants. **a** Carotenoid levels determined by HPLC analysis of T4 leaf tissues harvested from three independent transgenic plants for each construct. **b** Chlorophyll absorbance in the same leaves as in **a** measured by spectrophotometry. NT represents non-transgenic rice plants (*Oryza sativa* L. cv. Ilmi). All data are represented as the mean ± SE of three independent measurements. Statistical analyses were performed using a two-tailed Student’s *t*-test, and significant differences were indicated by a *p* value (** *p* < 0.01 and *** *p* < 0.001). Different varieties of transgenic plants are represented in different colors, such as green and light-green in the bar graph, and the X-axis labeling consists of three independent-transgenic plant lines

Firstly, we analyzed the carotenoid and chlorophyll contents in leaves of T4 generation homozygous transgenic lines using high-performance liquid chromatography (HPLC), and the data are shown in Table S1 (Additional file 4). Compared to NT leaf controls, the total content of carotenoids was slightly reduced by 20% in the *PGD1::OsDXR* lines with the statistical significance, and by 10% in the *PGD1::OsDXS2* lines (Fig. 4a). We also measured the contents of chlorophyll a and b in the same tissues, and the data are shown in Table S2 (Additional file 4). The content of total chlorophylls was slightly reduced by 11% in the *PGD1::OsDXS2* lines and by 15% in the *PGD1::OsDXR* lines (Fig. 4b) with the statistical significance. Taken together, the results show that the contents of carotenoids and chlorophylls are slightly reduced rather than increased by the overexpression of *OsDXS2* or *OsDXR* in rice leaves. We also tried to determine if the reduction of carotenoids and chlorophylls affected the leaf phenotypes, but no phenotypic changes were observed, suggesting that the reductions caused by OsDXS2 or OsDXR overexpression did not significantly affect leaf phenotypes.

### The organ-specific differential roles of OsDXS2 as a rate-limiting enzyme in rice seed carotenoid metabolism, compared to OsDXR

**Fig. 5:**
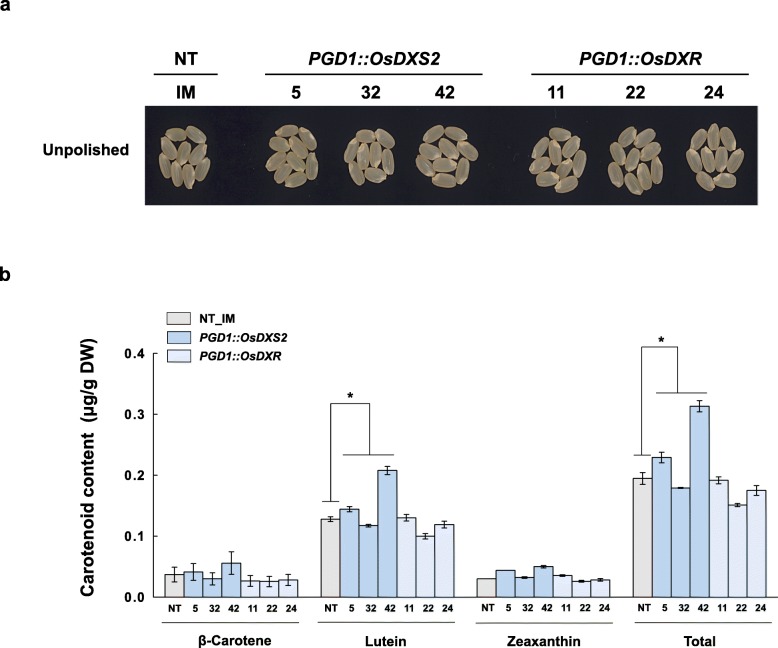
Color phenotypes and carotenoid levels in the seeds of *PGD1::OsDXS2* and *PGD1::OsDXR* transgenic plants relative to NT plants. **a** Mature seed colors in homozygous T4 generations were compared with NT (*Oryza sativa* L. cv. Ilmi) at 40 days after flowering (DAF) among the three independent transgenic plants for each construct before polishing because polished colors are indistinguishable from white. **b** Carotenoid levels determined by HPLC analysis in the same seeds as in **a**. All carotenoid content data are displayed as the mean value ± standard error (SE) of three independent measurements. Statistical analyses were performed using a two-tailed Student’s *t*-test (* *p <* 0.05). Different varieties of transgenic plants are represented in different colors, such as blue and light-blue in the bar graph, and the X-axis labeling consists of three independent-transgenic plant lines

**Fig. 6:**
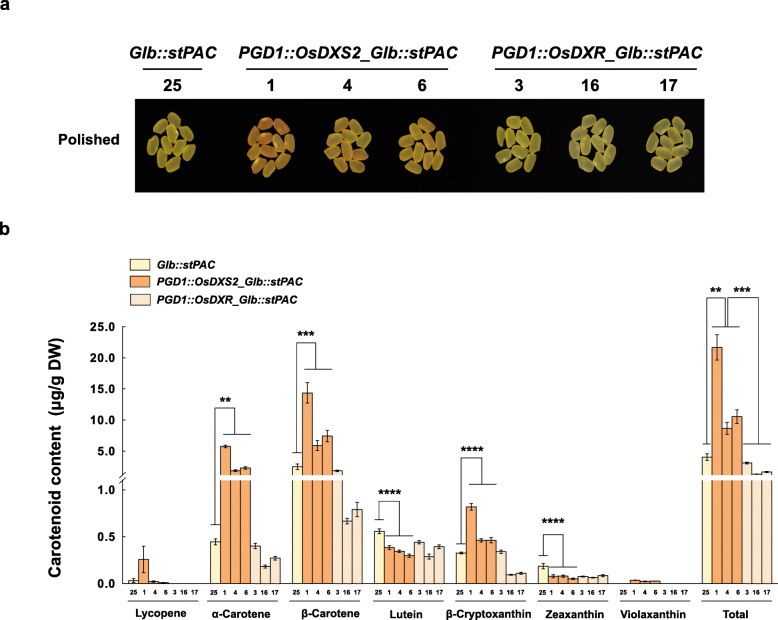
Color phenotypes and carotenoid levels in the seeds of *PGD1::OsDXS2_Glb::stPAC* and *PGD1::OsDXR_Glb::stPAC* relative to *Glb::stPAC* transgenic plants. **a** Mature seed colors in the homozygous T4 generation were compared among three independent transgenic plants for each construct with *stPAC* rice seeds 40 days after flowering (DAF) and after polishing. **b** Carotenoid levels measured by HPLC analysis in the same seeds as in **a**. All carotenoid content data are displayed as the mean value ± standard error (SE) of three independent measurements. Statistical analyses were performed using a two-tailed Student’s *t*-test (** *p* < 0.01, *** *p* < 0.001 and **** *p* < 0.0001). NT is a non-transgenic wild type of *Oryza sativa* L. cv. Ilmi, different varieties of transgenic plants are represented in different colors, such as orange, light-orange and yellow in the bar graph, and the X-axis labeling consists of three independent-transgenic plant lines

We next examined whether the overexpression of either OsDXS2 or OsDXR affected seed carotenoid metabolism (Fig. 5). Compared to NT seeds, although no color or phenotypical changes were observed in the brown seeds (Fig. 5a), total carotenoid content was increased by 26% in the PGD1::OsDXS2 lines and decreased by 11% in the PGD1::OsDXR lines (Fig. 5b and Additional file 4: Table S3) with statistical significance even if the alterations are a little. Also, as shown in Fig. 6, the yellow color in *Glb::stPAC* seeds [32] was largely intensified to an orange color in the *PGD1::OsDXS2_Glb::stPAC* lines, but remained just yellow in the *PGD1::OsDXR_Glb::stPAC* lines (Fig. 6a). Compared to *Glb::stPAC* seeds, the total carotenoid contents of *PGD1::OsDXS2_Glb::stPAC* seeds were largely increased by 5.4-fold (Fig. 6b). Specifically, the contents of lycopene, α-carotene and β-carotene increased 8.7-fold, 13.1-fold, and 5.8-fold, respectively, while lutein and zeaxanthin decreased 0.7-fold and 0.4-fold, respectively (Additional file 4: Table S3). In contrast, the overexpression of *OsDXR* did not enhance carotenoid accumulation in either the *PGD1::OsDXR* or *PGD1::OsDXR_Glb::stPAC* seeds (Fig. 6b), indicating the differential roles of OsDXR compared with the positive roles of OsDXS2 in the enhancement of seed carotenoid metabolism.

Interestingly, the level of total xanthophylls increased 6.4-fold, whereas both α-carotene and β-carotene contents were greatly enhanced by 315.3-fold in *PGD1::OsDXS2_Glb::stPAC* seeds compared with NT. This suggests that the hydroxylation step to convert carotenes into xanthophylls, which is one of the rate-limiting steps in carotenoid metabolism, is tightly regulated to maintain a basal level of xanthophylls in rice seeds.

### *OsDXS2*- or *OsDXR*-mediated transcriptional alteration of intrinsic carotenogenetic genes in rice leaves and seeds

**Fig. 7:**
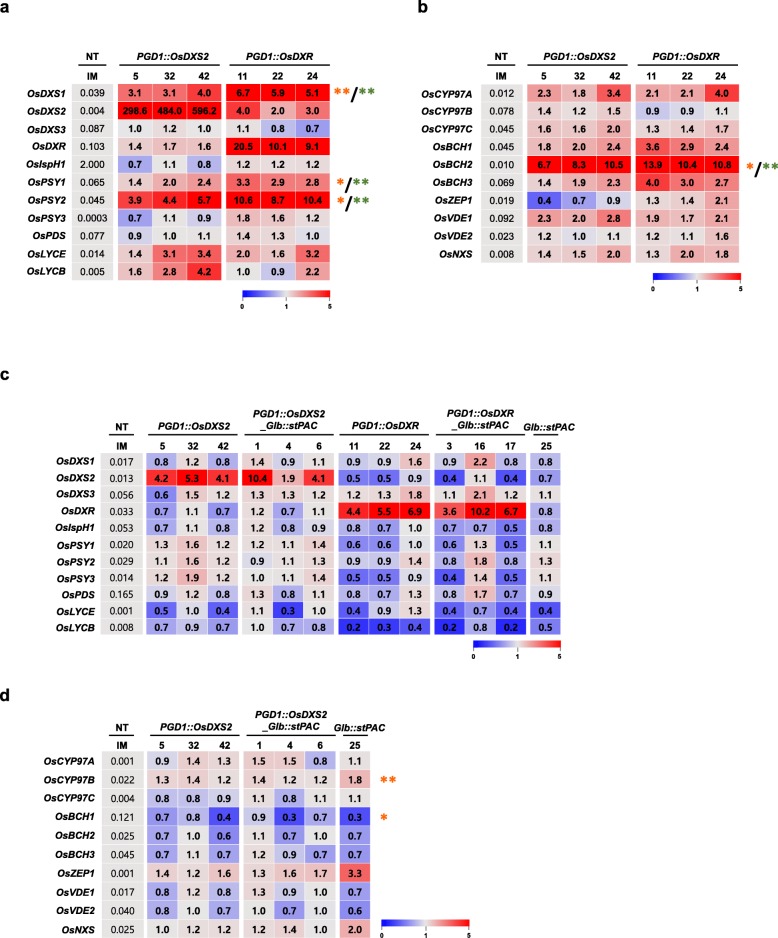
Expression profiles of carotenoid biosynthetic genes in leaves and seeds among three independent transgenic plants for each *OsDXS2* and *OsDXR* construct relative to non-transgenic (NT) and *stPAC* plants. In the 10-day-old leaves, the expression patterns of 11 rice genes, including five that are involved in the MEP pathway and six carotene biosynthetic genes (**a**) and 10 rice xanthophyll biosynthetic genes (**b**) are presented as the fold-change values (2^−ΔΔCt^) compared to the ΔCt values of NT, and the qRT-PCR data are listed in Additional file 4: Table S4. Statistical analyses of expression levels were performed using a two-tailed Student’s *t*-test (*p < 0.05 and ** *p* < 0.01). The *t*-test results are represented as an orange color (*) and a green color (*) for the significant effects of OsDXS2 and OsDXR enhancements, respectively. Also, the expression patterns of the same gene sets were analyzed in the mature seeds 40 days after flowering (DAF). The expression data of 11 rice genes, including five that are involved in the MEP pathway and six carotene biosynthetic genes (**c**) and 10 rice xanthophyll biosynthetic genes (**d**) are presented as the fold-change values (2^-ΔΔCt^) compared to the ΔCt values of NT, and all qRT-PCR data are listed in Additional file 4: Table S5–S6. The ΔCt values of NT are listed in the gray colored boxes, and all fold-change values (2^-ΔΔCt^) are presented with the corresponding heatmap graphs. The color key of the heatmap graph is presented under each graph, and the accession numbers of genes and primer sequences are listed in Additional file 4: Table S8

The transcript levels of the intrinsic genes involved in MEP, carotene, and xanthophyll pathways were determined in the leaves and seeds of NT and transgenic plants, and all expression patterns were visualized using a heatmap graph that represented the fold changes (Fig. 7). The expression data are shown in Additional file 4: Table S4-S6. As shown in Fig. 7a and b, the expression of endogenous carotenogenetic genes in leaves was mostly increased, as indicated by the red-color in the heatmap. The expression levels of these genes were further statistically analyzed using a two-tailed Student’s *t*-test, and the statistical significances were indicated with orange or green asterisks (Fig. 7a and b). The expression of the genes encoding DXS, PSY, and BCH enzymes were significantly increased following the overexpression of either *OsDXS2* or *OsDXR* (* *p* < 0.05 and ** *p* < 0.01). Of them, the levels of *OsDXS1*, *OsPSY2,* and *OsBCH2* were increased by 3.1-fold to 13.9-fold in both *OsDXS2* and *OsDXR* overexpressed transgenic leaves, suggesting that they might be associated with the enhanced activities of OsDXS2 and OsDXR in rice leaves. Interestingly, the endogenous expression of *OsDXS1* and *OsDXS2* increased up to 6.7-fold and 4.0-fold, respectively, following the overexpression of *OsDXR*, but the enhanced activity of OsDXS2 did not cause a significant increase in *OsDXR* expression, even though the expression of the *OsPSY2* gene was significantly increased up to 5.7-fold following the overexpression of *OsDXS2* (Fig. 7a). These results demonstrate the differential roles of DXS and DXR genes in rice leaves. However, the significant increases in gene expression were not proportional to the metabolic changes, considered with a slight reduction in the amount of carotenoids and chlorophylls in the leaves of the *PGD1::OsDXS2* and *PGD1::OsDXR* plants.

Twelve genes involved in the MEP and carotene biosynthetic pathways were also transcriptionally compared among seeds from five transgenic plants of *PGD1::OsDXS2*, *PGD1::OsDXS2_Glb::stPAC*, *PGD1::OsDXR*, *PGD1::OsDXR_Glb::stPAC,* and *Glb::stPAC,* and NT rice plants (Fig. 7c, Additional file 4: Table S5). In contrast to leaves, the expression of the genes encoding DXSs and PSYs were not highly upregulated by the overexpression of either *OsDXS2* or *OsDXR* and altered slightly without statistical significance in the seeds of *PGD1::OsDXS2* and *PGD1::OsDXR,* as shown by the faint pink and blue colors on the heatmap (Fig. 7c). The expression analysis of the biosynthetic genes involved in xanthophyll metabolism in the seeds of *PGD1::OsDXS2, PGD1::OsDXS2_Glb::stPAC* and *Glb::stPAC* showed that the expression patterns of *OsCYP97B* (a P450-type α-carotene hydroxylase gene) and *OsBCH1* (a β-carotene hydroxylase-1 gene) were significantly upregulated and downregulated, respectively, even though the expressions only changed by 2-fold (Fig. 7d). Therefore, these small changes in the expression patterns were inconsistent with the 73.8-fold and 315.3-fold increases observed in the carotenoid contents in *Glb::stPAC* and *PGD1::OsDXS2_Glb::stPAC* seeds, respectively, compared to NT seeds. In other words, the strong enhancement of carotenoid accumulation is quite dependent on the addition of transgenes rather than changes in endogenous carotenogenic gene expression.

Collectively, the expression of endogenous carotenogenic genes was differentially regulated by either *OsDXS2* or *OsDXR* overexpression in an organ-specific manner, but there was no significant correlation between their expression level and subsequent metabolic changes. These results suggest that the large enhancement of carotenoid accumulation is mediated by the stepwise addition of *OsDXS2* and *stPAC* genes, but not largely dependent on any changes in the expression of intrinsic carotenogenic genes.

## Discussion

In this study, we investigated the differential roles of rice DXS and DXR as the first two enzymes of MEP pathway on rice carotenoid metabolism. The content of total seed carotenoids was increased by 26% in the *PGD1::OsDXS2* lines compared with NT plants and was considerably increased on average by 3.4-fold (5.4-fold maximum) in the *PGD1::OsDXS2_Glb::stPAC* lines compared with the *Glb::stPAC* lines, but in contrast, decreased by 11% in the *PGD1::OsDXR* lines and by 50% in the *PGD1::OsDXR_Glb::stPAC* lines compared to NT plants and *Glb::stPAC* rice lines, respectively (Fig. 5 and Fig. 6). Considering that the phytoene biosynthesis is quite limited in rice endosperm [28], the large enhancement of carotenoid accumulation in *PGD1::OsDXS2_Glb::stPAC* seeds suggests that OsDXS2 functions as a rate-limiting enzyme supplying IPP/DMAPPs to carotenoid metabolism, even though total carotenoid content in *PGD1::OsDXS2* seeds was a little changed to increase just by 26%. However, in rice leaves, total carotenoids were reduced by an average of 10% in the *PGD1::OsDXS2* lines and by 20% in the *PGD1::OsDXR* lines. Further, total chlorophylls were also reduced by an average of 11% in *PGD1::OsDXS2* and 15% in *PGD1::OsDXR* compared to NT plants (Fig. 4). These results suggest that OsDXS2 functions as a rate-limiting enzyme just only in seed carotenoid metabolism but not in leaves, whereas OsDXR is a rate-limiting enzyme in neither leaves nor seeds.

In Arabidopsis leaves, both AtDXS and AtDXR are rate-limiting enzymes in the biosynthetic pathways that produce carotenoid and chlorophyll [4, 5], however, the differential roles of plant DXS and DXR enzymes have also been consistently reported in several plant species. In carrot, the overexpression of *AtDXR* failed to increase the content of total carotenoids in both tissues, while *AtDXS* overexpression did increase the content of total carotenoids in both leaves and roots by modifying the expression of *PSY1* and *PSY2* genes [7]. In lavender, the overexpression of *AtDXS* did not affect the carotenoid and chlorophyll contents in leaves but did significantly increase the essential oil monoterpene [8]. In contrast, *AtDXR* did not mediate any changes in essential oil, carotenoid or chlorophyll contents [9]. These studies clearly demonstrate that the rate-limiting functions of the DXR enzyme may not be consistent and the rate-limiting functions of the DXS enzyme could vary between plant species or even different tissues.

In this study, the expression profiling of rice carotenogenic genes involved in the MEP, carotene, and xanthophyll pathways showed that their expression patterns were not proportional to the changes in carotenoid metabolism in the leaves and seeds of rice transgenic plants. The expression of carotenogenic genes, such as *OsDXS1*, *OsPSY1*, *OsPSY2,* and *OsBCH2,* were upregulated by the enhancement of either *OsDXS2* or *OsDXR* genes in leaves compared to the reduction of the carotenoid and chlorophyll content, and there were only slight changes in the expression of most carotenogenic genes, even though there was a large increase in the seed carotenoid accumulation (Fig. 7). In contrast to our results, the overexpression of a DXS-encoding gene in tomato fruits and potato tubers increased the expression of the endogenous PSY-encoding genes and subsequently caused a large enhancement of carotenoid accumulation [34, 35]. Similar results have been observed in carotenoid metabolism in carrot leaves and roots [7]. These results show that the DXS-mediated increase of carotenoid and chlorophyll contents is correlated with an increase in *PSY* gene expression. However, a positive correlation was not observed in rice leaves. Specifically, the expression of *OsPSY1* and *OsPSY2* genes were preferentially upregulated compared to the reduction of carotenoid content in leaves, and were not altered in rice seeds, even though there was an increase in carotenoid content. Similarly, in tomato fruits, the expression of the DXS-, PSY-, and PDS-encoding genes are preferentially downregulated compared to the enhancement of β-carotene [34], and in *Bixa orellana* L., the carotenoid content was increased by the salt stress treatment, but the expression of carotenogenic genes were non-proportionally downregulated [36]. These results suggest the possibility that the transcriptional expression of the intrinsic carotenogenic genes, such as the DXS- and PSY-encoding genes, might not be proportional to the expression levels of their encoded-proteins, and the protein stability of the intermediate biosynthetic enzymes might be equally as important as the enhanced activity of rate-limiting enzymes for the enhancement of carotenoid accumulations.

In *Arabidopsis*, AtDXS proteins have been reported to be post-translationally regulated by chloroplast biogenesis 6 (CLB6, a hydroxy-2-methyl-butenyl 4-diphosphate reductase), Hsp100 chaperones, and several Clp proteases [37], and the proteostasis of *Arabidopsis* PSY proteins are controlled directly by the Clp protease and ORANGE protein [38]. In *Catharanthus roseus*, the stability of DXR proteins is highly dependent on Clp protease-mediated degradation [39], and the enzyme activity of PDS proteins is more stably sustained in the β-carotene-enhanced tomato fruits, even if its transcripts are downregulated [34]. Similarly, our studies showed that the seed carotenoid accumulations could be largely increased without any proportional upregulation of the intermediate biosynthetic genes, except the enhancement of the rate-limiting enzymes, such as DXS and PSY, suggesting that seed carotenoid metabolism might be preferentially controlled by the stability or activity of the carotenogenic enzymes, rather than their transcript levels.

**Fig. 8:**
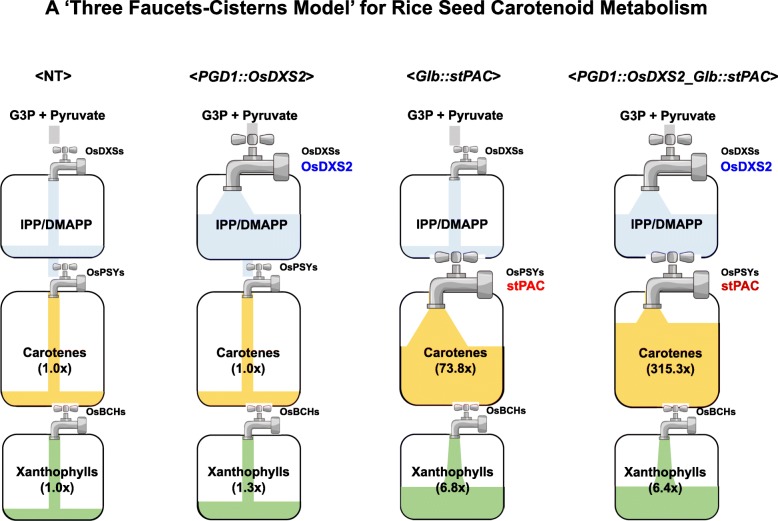
Proposed “Three Faucets and Cisterns Model” as a carotenoid accumulation mechanism for the rice seed system. The rate-limiting key enzymes are displayed as “Faucets,” and the biosynthetic and accumulation machinery are shown as squares representing the “Cisterns.” The metabolite pools of G3P/pyruvate, IPP/DMAPP, carotenes, and xanthophylls are presented as four colors of gray, sky-blue, yellow, and green, respectively. *OsDXS2* or *stPAC* transgenes are signified as blue or red, respectively. Carotene components are composed of lycopene, α-carotenes, and β-carotenes, and xanthophyll components contain lutein, β-cryptoxanthin, zeaxanthin, and violaxanthin

Collectively, we propose a “Three Faucets and Cisterns Model” to describe rice seed carotenoid metabolism (Fig. 8). The term “Three Faucets” represents the three rate-limiting steps involving DXS, PSY, and BCH enzymes that supply restricted levels of precursors from upstream to downstream pathways. The intermediate carotenoid biosynthetic machinery between two faucets is regarded as the “Cistern” that fills with particular carotenoid metabolites representing IPP/DMAPP, carotenes, and xanthophylls in our model, and whose capacity is assumed to be determined only by the status of active proteins (Fig. 8). By following the “Three Faucets and Cisterns Model,” the overexpression of OsDXS2 in *PGD1::OsDXS2* seeds might turn on the first “Faucet” to increase the metabolic flux into the first “Cistern,” and the carotenoid content could slightly increase (by 1.3-fold) as much as the capacity of the second “Faucet” (rice PSY genes), which is maintained at a basal level in rice seeds. Also, by the stepwise addition of *OsDXS2* and *stPAC*, both the first and second “Faucets” are turned on to increase the metabolic flux from the first “Cistern” to the second “Cistern,” and these consecutive metabolic streams could increase protein stability to enhance the capacity of the “Cisterns” and enable the large enhancement of α/β-carotene accumulation by 315.3-fold without any changes in the expression of “Cistern” genes. In the case of *Glb::stPAC* seeds, the second “Faucet” (stPAC) was turned on without any enhancement of the first “Faucet,” but the carotenoid content was increased by 73.8-fold. In contrast, in *PGD1::OsDXR* seeds, the enhancement of OsDXR regarded as a component of the first “Cistern” did not increase carotenoid accumulation, clearly demonstrating the differential functions between the “Faucet” and “Cistern.” In other words, the enhancement of the second “Faucet” (stPAC) could increase the metabolic flux from the first “Cistern” to the second “Cistern,” but the increase of a “Cistern” component (OsDXR) might fail to affect the metabolic flux from the upstream steps to the second “Faucet,” therefore any enhancement of carotenoid accumulation was not observed in rice seeds. Finally, the contents of xanthophylls were gradually increased by the stepwise addition of either *OsDXS2* or *stPAC* genes from 1.3-fold to 6.8-fold in rice seeds, but no additional increase was observed in *PGD1::OsDXS2_Glb::stPAC* seeds, whose carotene content was increased by 4.3-fold compared to *Glb::stPAC* seeds, suggesting that it might be due to the maximum capacity of the third “Faucet.”

## Conclusion

In this study, the differential roles of OsDXS2 and OsDXR in carotenoid metabolism of rice leaves and seeds were investigated, and the changes in expression of carotenogenic genes were measured. The results suggest that OsDXS2 is a rate-limiting enzyme supplying IPP/DMAPPs to carotenoid metabolism in rice seeds but not in rice leaves, whereas no OsDXR has function as a rate-limiting enzyme in leaves or seeds. The expression profiling of the carotenogenic genes showed non-proportional correlations between the changes in gene expression and the metabolic changes, suggesting that the biosynthetic activity of carotenoid intermediate metabolites could be enhanced without any increases in transcript levels. Taken together, these differential roles of OsDXS2 and OsDXR are suggested to occur in a rice plant-specific pattern, and the “Three Faucets and Cisterns Model” was proposed to describe carotenoid metabolism in rice seeds. Finally, our studies provide useful information for designing fine-tuned strategies of carotenoid metabolic engineering in rice plants.

## Methods

### Plant materials and growth conditions

Mature seeds of Korean rice (*Oryza sativa* L. cv. Ilmi) were germinated and grown in soil in a greenhouse under the conditions of a16 h light/8 h dark cycle at 28 °C. Rice seeds obtained from the National Institute of Agricultural Sciences (South Korea) were used for *Agrobacterium*-mediated transformation and expression analyses of DXS genes. Organ-specific samples were harvested at different stages and used for quantitative real-time (qRT)-PCR.

T0 generation transgenic rice plants were first grown in a growth chamber, and then after transplanting them into the soil, T1 and T2 generations were grown in the greenhouse under the same conditions of a 16 h light/8 h dark cycle at 28 °C and in the field during the summer season until the T4 seed generation. All field studies were performed following local legislation with the permissions of the Rural Development Administration (South Korea). After the sterilization of T4 seeds with 70% ethanol and 2% sodium hypochlorite, 10-day-old seedlings were grown in a room maintained at 28 °C with a 12 h light/12 h dark cycle and under 70–90% relative humidity. These seedlings were used for qRT-PCR and metabolite analysis with T4 seeds, which were harvested at full maturity 40 days after flowering (DAF). The seed endosperm color was visually compared after dehusking (TR-200 Electromotion Rice Husker, Kett, Tokyo, Japan) and polishing (Pearlest Polisher, Kett).

### Vector construction

The coding regions, including the open reading frames of *OsDXS2* (Os07g09190) and *OsDXR* (Os01g01710), were amplified from the total RNA of 10-day-old seedlings using the gene-specific primer pairs F1/R1 and F2/R2, respectively. Each amplicon was further amplified using the universal *attB* primer pair and introduced into the *pDONR221* vector by recombination using the Gateway® BP Clonase® II Enzyme Mix (Invitrogen, Waltham, MA). The resultant subclones were recombined with the vector *p600-PGD1* (Seoul National University, Pyeongchang Korea), which contained a rice phosphogluconate dehydrogenase 1 (PGD1) promoter for constitutive expression in rice plants, [40] using the Gateway® LR Clonase® II Enzyme Mix (Invitrogen). This resulted in the generation of *pPGD1::OsDXS2* and *pPGD1::OsDXR* vectors for rice transformation (Fig. 1b).

To introduce entire *PGD1::OsDXS2::PinII* or *PGD1::OsDXR::PinII* cassettes into the region of the PGD1 promoter and the PinII terminator into the *stPAC* expression cassette, which produces *β*-carotene under the control of a rice endosperm-specific globulin (Glb) promoter [32], they were individually PCR-amplified with the F3/R3 primer pair and then cloned into a *pGlb::stPAC* vector using the *Xho*I site, yielding either *pPGD1::OsDXS2_Glb::stPAC* or *pPGD1::OsDXR_Glb::stPAC* for rice transformation (Fig. 1b). All PCRs were performed using a PrimeSTAR® HS DNA Polymerase (Takara, Shiga, Japan). All primers are shown in Fig. 3b and their sequences are listed in Additional file 4: Table S7.

### Rice transformation and selection of transformants

For overexpression of *OsDXS2* or *OsDXR*, the *pPGD1::OsDXS2*, *pPGD1::OsDXR*, *pPGD1::OsDXS2_Glb::stPAC,* and *pPGD1::OsDXR_Glb::stPAC* final vectors, were individually introduced into *Escherichia coli* DH5α and then *Agrobacterium tumefaciens* LBA4404 harboring *pSB1* plasmids through tri-parental mating [41]. After co-cultivation with embryogenic calli that were differentiated from the mature seeds of rice (*O. sativa* L. cv. Ilmi), the putative transgenic plantlets were generated using selection media containing phosphinothricin (4 mg/L) and cefotaxime (500 mg/L) under growth chamber conditions, according to a previously published method [42].

Genomic DNA was purified from leaf tissues using a DNeasy Plant Mini Kit (Qiagen), after grinding with a TissueLyser II (Qiagen), and PCR-amplified using a MightyAmp® DNA Polymerase (Takara) following the manufacturer’s instructions. The positive transgenic plants were first screened at the T0 leaf generation by PCR using the primer pairs F4/R4 for *OsDXS2*, F4/R5 for *OsDXR*, and F6/R6 for *stPAC* transgenes. Three selected lines were further verified using the same PCR protocols at the T3 leaf generation after a segregation test on phosphinothricin (4 mg/L) to assess the homozygosity of T-DNA. To examine the transgene copy number in rice genomes, TaqMan real-time PCR was performed using the primer set NF/NR, a customized probe NP labeled with a 6-carboxyfluorescein dye to detect a *Nos* terminator on the *BAR* cassette (*35S::Bar::Nos*), and the customized VIC dye-labeled α-tubulin probe (Os11g14220), as an internal reference (Assay ID: Os03643486_s1; Applied Biosystems, Foster City, CA). The PCR was carried out using a TaqMan Gene Expression Master Mix (Applied Biosystems), and fluorescence was measured with the CFX Connect™ Real-Time System (Bio-Rad, Richmond, CA) relative to the value of 1 copy in the homozygous T3 generation of the *stPAC 25* rice line [32] using PCR conditions, as previously described [43]. The sequences of primers and probes used in the genomic DNA analysis are listed in Additional file 4: Table S7.

### Quantification of carotenoids and chlorophylls

Leaf and seed samples of rice plants were prepared for carotenoid extraction, as previously described [43]. For HPLC analysis, the analytical samples were prepared by dissolving in dichloromethane/methanol 50:50 (v/v) following the addition of β-apo-8′-carotenal (0.05 mL of 25 μg/mL, Sigma-Aldrich Chemical Co, St. Louis, MO) as an internal standard, separating into layers with hexane (1.5 mL), and desiccating under liquid nitrogen. Carotenoids were then separated with a YMC ODS C-30 column (3 μm, 4.6 × 250 mm; YMC Europe, Germany) by an Agilent 1100 Series HPLC system (Agilent, Santa Clara, CA) equipped with a photodiode array detector under elution conditions, as previously described [43]. The chromatograms were generated at 472 nm for lycopene and 450 nm for the other compounds, including α-carotene, (all-*E*)-*β*-carotene, 9*Z*-*β*-carotene, 13*Z*-*β*-carotene, *β*-cryptoxanthin, lutein, violaxanthin, and zeaxanthin. The quantification was determined from the HPLC peak areas relative to the external standard calibration curves of the carotenoid standards, which were purchased from CaroteNature (Lupsingen, Switzerland). The amount of *β*-carotene was determined as the sum of (all-*E*)-*β*-carotene, 9*Z*-*β*-carotene, and 13*Z*-*β*-carotene.

To extract chlorophylls, 10 mg of the fresh leaf powder of rice plants were mixed with 1 mL of 100% methanol and incubated at 70 °C for 30 min with shaking (500 rpm) using a Thermomixer Comfort (model 5355, Eppendorf AG, Hamburg, Germany). After centrifuging at 800 *g* for 10 min at 4 °C, the absorbance of the supernatant was measured at 666 nm and 653 nm in a spectrophotometer (Optizen Pop, Mecasys Co, Daejeon, Korea). The contents of chlorophyll *a* and *b* were calculated using Wellburn’s formula (1994).

### Quantitative real-time PCR

Total RNA was purified from the leaf, root, and floret tissues at nine weeks (vegetative stage) and three months (reproductive stage) to compare the expression patterns of *OsDXS1*, *OsDXS2*, *OsDXS3,* and *OsDXR* genes. Total RNA was also purified from the leaves of 10 day-old seedlings and from the unpolished mature seeds 40 DAF to profile the expression patterns of carotenogenic genes in each transgenic plant. The frozen powder (100 mg) samples were resolved in the PureLink® Plant RNA Reagent (Invitrogen) with DNase I (Qiagen, Hilden, Germany) for removal of remnant genomic DNA contamination. The 1st cDNA was synthesized using the AccuPower® RT Premix (Bioneer, Daejeon, Korea) and mixed with the SYBR Green Real-time PCR master mix (Bio-Rad). All reactions were performed with a CFX Connect™ Real-Time System (Bio-Rad) according to the manufacturer’s instructions and under the following conditions: 1 cycle of 3 min at 95 °C, 40 cycles of 15 s at 95 °C, and 1 cycle of 30 s at 60 °C. The qRT-PCR for transgene expression was performed with the gene-specific primer pairs F7/R7 for *OsDXS2*, F8/R7 for *OsDXR*, and F9/R9 for *stPAC*, as indicated in Fig. 1b and Additional file 4: Table S7. The transcripts of the 22 genes involved in rice carotenogenesis were examined by qRT-PCR using gene-specific primers. Gene names, accession numbers, primer sequences, and product sizes are detailed in Additional file 4: Table S8. To normalize the amount of RNA, all qRT-PCR values were calculated relative to the rice *ubiquitin 5* gene (Os01g22490), which was amplified using the U5F/U5R primer pair [44].

### Statistical analysis

All experiments were performed with a minimum of three biological replicates, and the results were expressed as the mean ± standard error (SE). Any statistically significant differences between the two groups were determined using a two-tailed Student’s *t*-test. A *p* value < 0.05 was considered to be statistically significant.

## Supplementary information

**Additional file 1: Figure S1.** Alignment of deduced amino acid sequences among plant type II deoxyxylulose 5-phosphate synthase (DXS) proteins.

**Additional file 2: Figure S2.** Alignment of deduced amino acid sequences among plant deoxyxylulose 5-phosphate reductoisomerase (DXR) proteins.

**Additional file 3: Figure S3.** Genomic DNA analyses to verify the integration and copy number of transgenes in the rice genome.

**Additional file 4: Table S1.** Carotenoid content and composition in the leaves of transgenic rice plants. **Table S2.** Chlorophyll content in the leaves of transgenic rice plants. **Table S3.** Carotenoid content and composition in mature seeds of transgenic rice plants. **Table S4.** Expression profiles of structural genes related to the biosynthesis of carotenoids in rice leaves. **Table S5.** Expression profiles of structural genes related to the supplement of substrates into the biosynthesis of rice seed carotenes. **Table S6.** Expression profiles of structural genes related to the xanthophyll biosynthesis in rice seeds. **Table S7.** The primer list used in vector construction and transgene analysis. **Table S8.** The primer list used in expression analysis of rice genes.

## Data Availability

The material and datasets used and/or analyzed during the current study are available from the corresponding author upon reasonable request.

## Abbreviations

BCH: A β-carotene hydroxylase
CYP97B: A P450-type-carotene hydroxylase gene
DMAPP: A dimethylallyl pyrophosphate
DXP: A deoxyxylulose 5-phosphate
DXR: A deoxyxylulose 5-phosphate reductoisomerase
DXS: A Deoxyxylulose 5-phosphate synthase
Glb: A rice endosperm-specific globulin
IPP: A isopentenyl pyrophosphate
MEP: A methylerythritol 4-phosphate
MVA: A mevalonic acid
PDS: A pytoene desaturase
PGD1: A rice phosphogluconate dehydrogenase 1
PSY: A pytoene synthase
stPAC: A *synthetic phytoene synthase:2A:CrtI*
