# Supplementary material for: The organ-specific differential roles of rice DXS and DXR, the first two enzymes of the MEP pathway, in carotenoid metabolism in Oryza sativa leaves and seeds
Source: BMC Plant Biol. 2020 Apr 15;20:167. doi: 10.1186/s12870-020-02357-9 (PMC7161295; doi:10.1186/s12870-020-02357-9)
Supplement: Supplementary file 1 — Additional file 1: Figure S1. Alignment of deduced amino acid sequences among plant type II deoxyxylulose 5-phosphate synthase (DXS) proteins. [file 12870_2020_2357_MOESM1_ESM.pptx]

## Slide 1
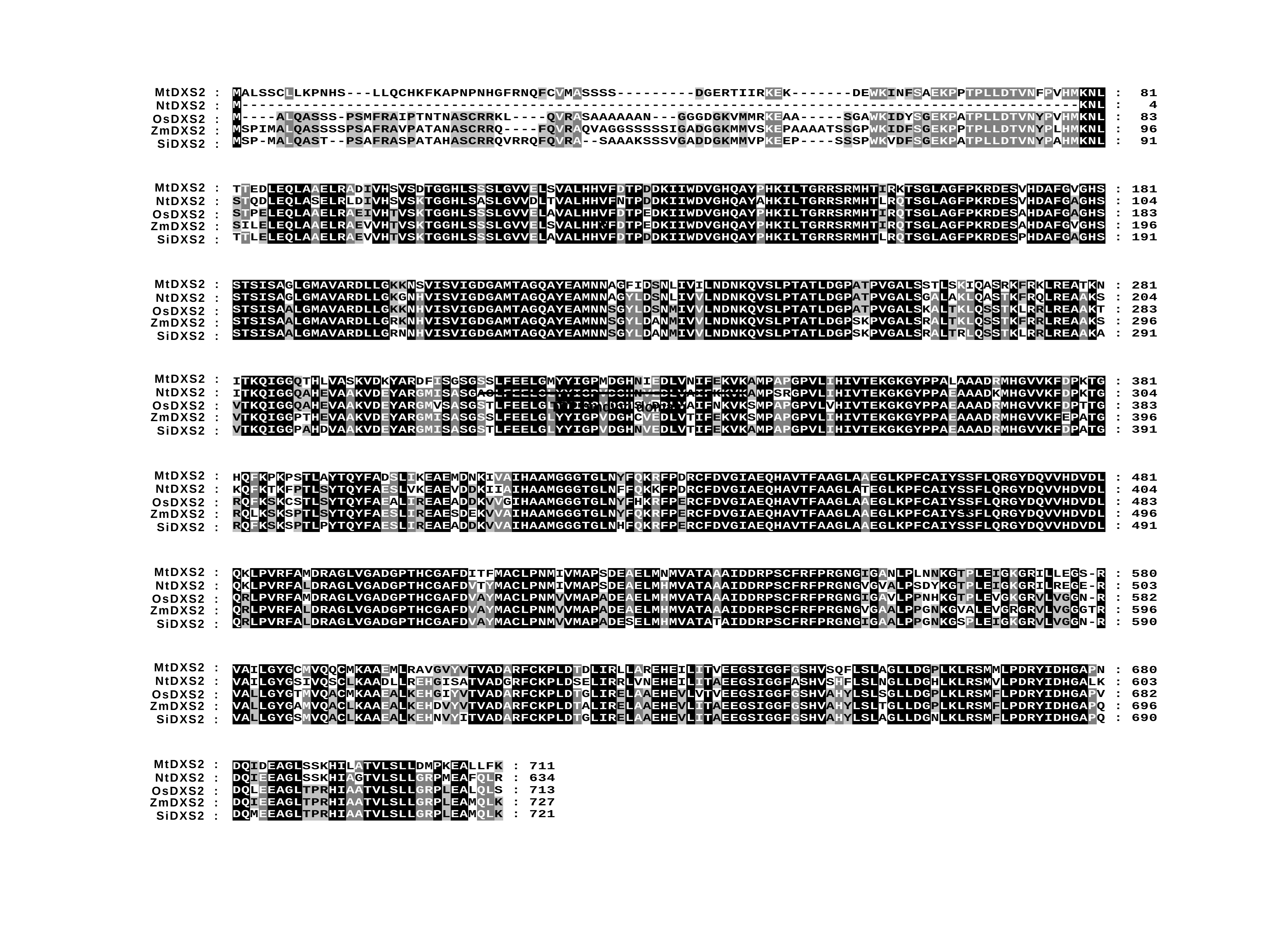

MtDXS2 :
NtDXS2 :
OsDXS2 :
ZmDXS2 :
SiDXS2 :
MtDXS2 :
NtDXS2 :
OsDXS2 :
ZmDXS2 :
SiDXS2 :
MtDXS2 :
NtDXS2 :
OsDXS2 :
ZmDXS2 :
SiDXS2 :
MtDXS2 :
NtDXS2 :
OsDXS2 :
ZmDXS2 :
SiDXS2 :
MtDXS2 :
NtDXS2 :
OsDXS2 :
ZmDXS2 :
SiDXS2 :
MtDXS2 :
NtDXS2 :
OsDXS2 :
ZmDXS2 :
SiDXS2 :
MtDXS2 :
NtDXS2 :
OsDXS2 :
ZmDXS2 :
SiDXS2 :
MtDXS2 :
NtDXS2 :
OsDXS2 :
ZmDXS2 :
SiDXS2 :
*
TPP binding domain
*
