# Supplementary material for: The organ-specific differential roles of rice DXS and DXR, the first two enzymes of the MEP pathway, in carotenoid metabolism in Oryza sativa leaves and seeds
Source: BMC Plant Biol. 2020 Apr 15;20:167. doi: 10.1186/s12870-020-02357-9 (PMC7161295; doi:10.1186/s12870-020-02357-9)
Supplement: Supplementary file 2 — Additional file 2: Figure S2. Alignment of deduced amino acid sequences among plant deoxyxylulose 5-phosphate reductoisomerase (DXR) proteins. [file 12870_2020_2357_MOESM2_ESM.pptx]

## Slide 1
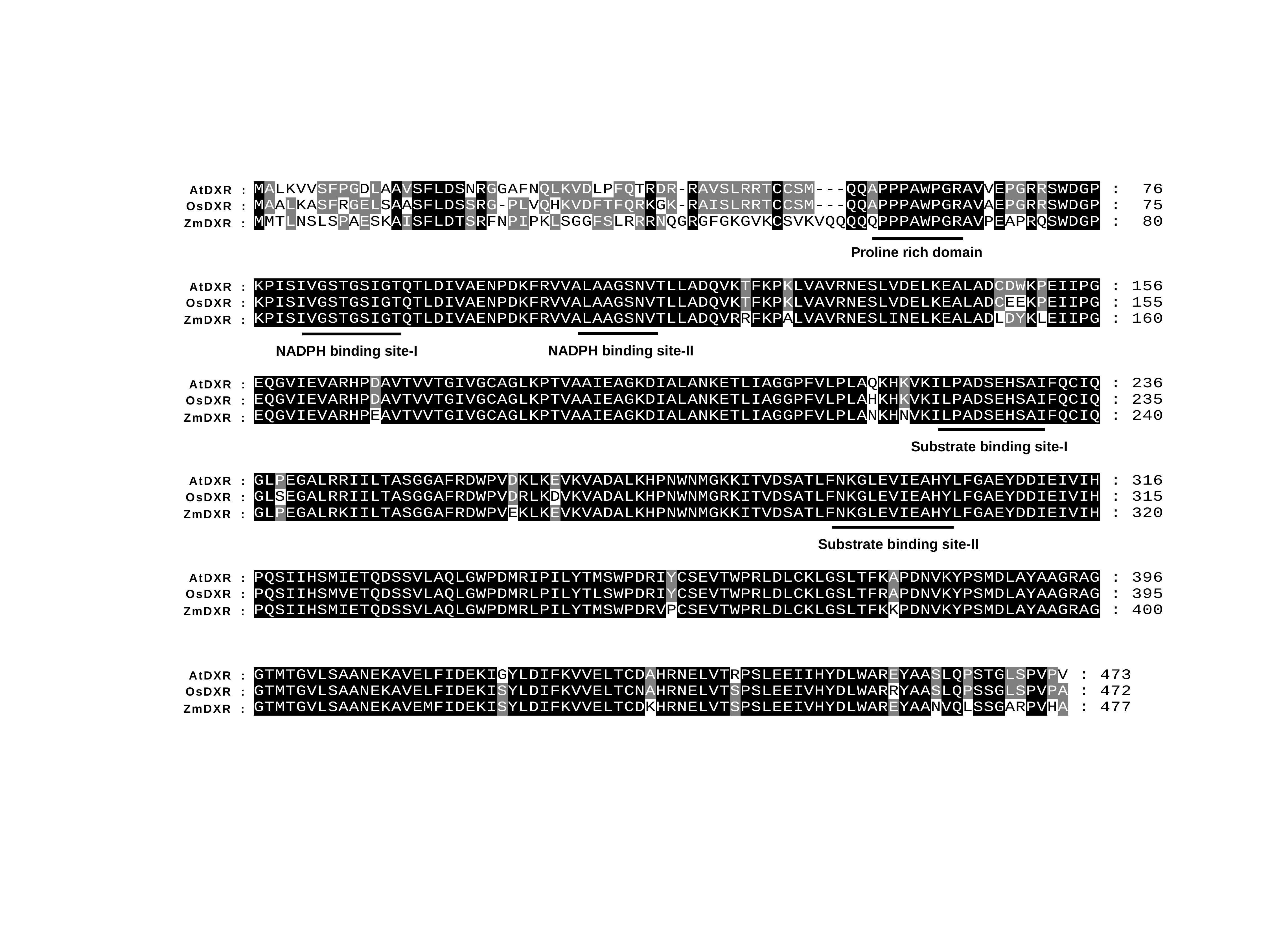

AtDXR :
OsDXR :
ZmDXR :
Proline rich domain
AtDXR :
OsDXR :
ZmDXR :
NADPH binding site-II
NADPH binding site-I
AtDXR :
OsDXR :
ZmDXR :
Substrate binding site-I
AtDXR :
OsDXR :
ZmDXR :
Substrate binding site-II
AtDXR :
OsDXR :
ZmDXR :
AtDXR :
OsDXR :
ZmDXR :
