# Supplementary material for: The organ-specific differential roles of rice DXS and DXR, the first two enzymes of the MEP pathway, in carotenoid metabolism in Oryza sativa leaves and seeds
Source: BMC Plant Biol. 2020 Apr 15;20:167. doi: 10.1186/s12870-020-02357-9 (PMC7161295; doi:10.1186/s12870-020-02357-9)
Supplement: Supplementary file 3 — Additional file 3: Figure S3. Genomic DNA analyses to verify the integration and copy number of transgenes in the rice genome. [file 12870_2020_2357_MOESM3_ESM.pptx]

## Slide 1
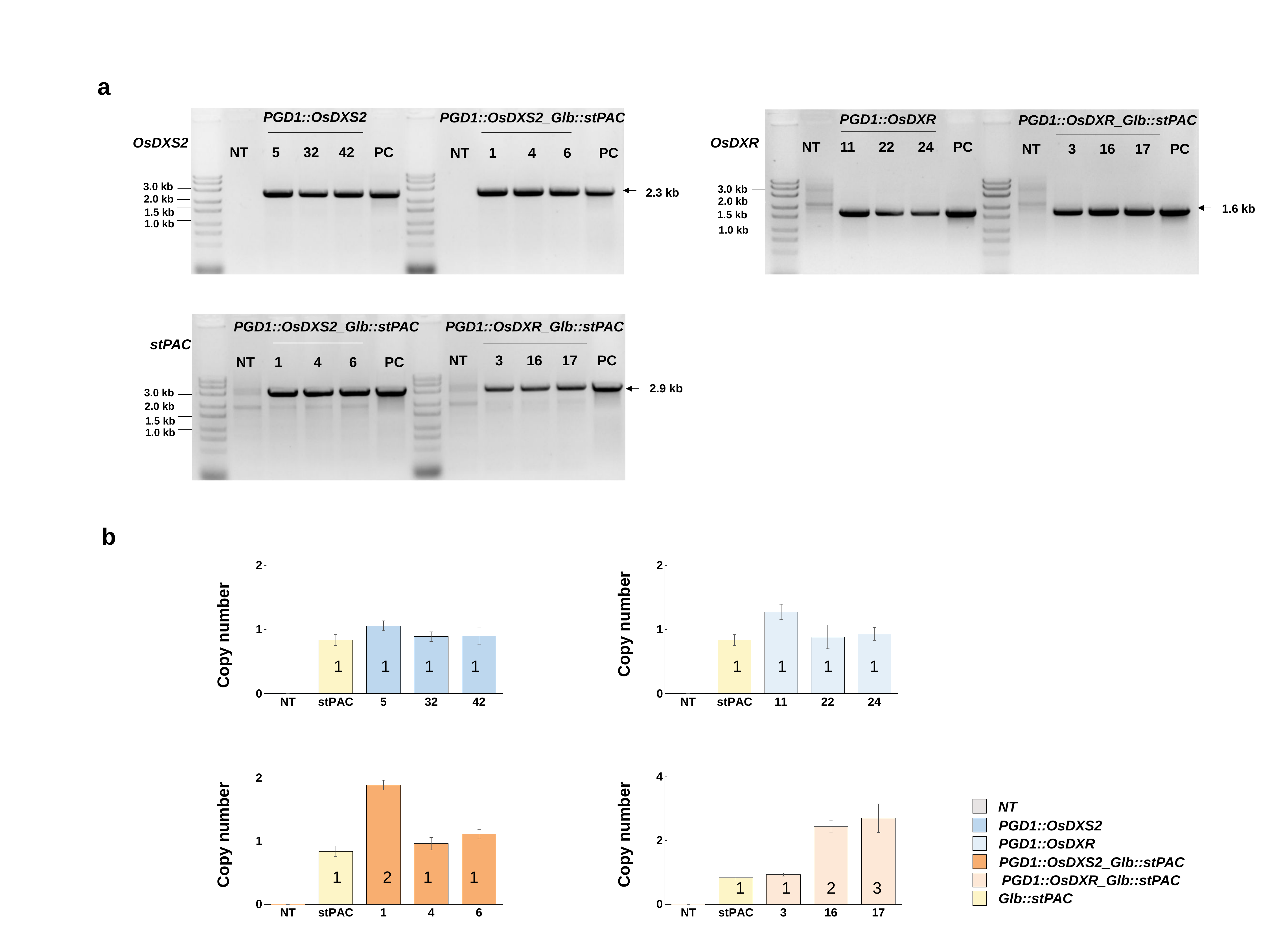

a
PGD1::OsDXS2
PGD1::OsDXS2_Glb::stPAC
PGD1::OsDXR
PGD1::OsDXR_Glb::stPAC
 OsDXS2
 OsDXR
 NT 11 22 24 PC
 NT 3 16 17 PC
 NT 5 32 42 PC
 NT 1 4 6 PC
3.0 kb
3.0 kb
2.3 kb
2.0 kb
2.0 kb
1.6 kb
1.5 kb
1.5 kb
1.0 kb
1.0 kb
PGD1::OsDXS2_Glb::stPAC
PGD1::OsDXR_Glb::stPAC
 stPAC
 NT 3 16 17 PC
 NT 1 4 6 PC
2.9 kb
3.0 kb
2.0 kb
1.5 kb
1.0 kb
b
### Chart
| Category | Expression |
|---|---|
| NT | 0.000442014271220705 |
| stPAC | 0.83624 |
| 5 | 1.06014225245381 |
| 32 | 0.889240325197565 |
| 42 | 0.893658032555404 |
### Chart
| Category | Expression |
|---|---|
| NT | 0.000442014271220705 |
| stPAC | 0.83624 |
| 11 | 1.27521102869169 |
| 22 | 0.88323271937779 |
| 24 | 0.930523120216578 |Copy number
Copy number
1
1
1
1
1
1
1
1
### Chart
| Category | Expression |
|---|---|
| NT | 0.000442014271220705 |
| stPAC | 0.83624 |
| 3 | 0.929678536290212 |
| 16 | 2.44102927940189 |
| 17 | 2.70483962268571 |
### Chart
| Category | Expression |
|---|---|
| NT | 0.000442014271220705 |
| stPAC | 0.83624 |
| 1 | 1.88432929879295 |
| 4 | 0.958249452603134 |
| 6 | 1.10992249468235 |NT
PGD1::OsDXS2
PGD1::OsDXR
PGD1::OsDXS2_Glb::stPAC
PGD1::OsDXR_Glb::stPAC
Glb::stPAC
Copy number
Copy number
1
2
1
1
1
1
2
3
