# Supplementary material for: The organ-specific differential roles of rice DXS and DXR, the first two enzymes of the MEP pathway, in carotenoid metabolism in Oryza sativa leaves and seeds
Source: BMC Plant Biol. 2020 Apr 15;20:167. doi: 10.1186/s12870-020-02357-9 (PMC7161295; doi:10.1186/s12870-020-02357-9)
Supplement: Supplementary file 4 — Additional file 4: Table S1. Carotenoid content and composition in the leaves of transgenic rice plants. Table S2. Chlorophyll content in the leaves of transgenic rice plants. Table S3. Carotenoid content and composition in mature seeds of transgenic rice plants. Table S4. Expression profiles of structural genes related to the biosynthesis of carotenoids in rice leaves. Table S5. Expression profiles of structural genes related to the supplement of substrates into the biosynthesis of rice seed carotenes. Table S6. Expression profiles of structural genes related to the xanthophyll biosynthesis in rice seeds. Table S7. The primer list used in vector construction and transgene analysis. Table S8. The primer list used in expression analysis of rice genes. [file 12870_2020_2357_MOESM4_ESM.docx]

**Table S1**. Carotenoid content and composition in the leaves of transgenic rice plants.

| **Plants** | **Line No.** | | **Carotenoid content (%)** | | | | | | | **Total carotenoid** | **β/α Ratio** |
| --- | --- | --- | --- | --- | --- | --- | --- | --- | --- | --- | --- |
|  |  |  | **Lycopene** | **α-Carotene** | **β-Carotene** | **Lutein** | **β-Cryptoxanthin** | **Zeaxanthin** | **Violaxanthin** |  |  |
|  |  |  | |  |  |  |  |  |  |  |  |
| **NT** | **IM** | N.D. | | 3.09±0.35 (1.4) | 129.70±2.41 (58.0) | 89.46±2.42 (40.0) | N.D. | 1.30±0.79 (0.6) | N.D. | 223.54±5.32 | 1.42±0.02 |
|  |  |  | |  |  |  |  |  |  |  |  |
| ***PGD1::OsDXS2*** | **5** | N.D. | | 2.86±0.11 (1.3) | 125.68±6.17 (58.3) | 84.84±1.95 (39.4) | N.D. | 2.02±0.20 (0.9) | N.D. | 215.41±8.26 | 1.45±0.04 |
|  | **32** | N.D. | | 2.58±0.18 (1.4) | 99.11±3.14 (55.3) | 75.55±1.09 (42.2) | N.D. | 1.99±0.08 (1.1) | N.D. | 179.23±3.41 | 1.29±0.04 |
|  | **42** | N.D. | | 2.56±0.08 (1.2) | 120.42±8.92 (57.9) | 83.63±4.50 (40.2) | N.D. | 1.38±0.28 (0.7) | N.D. | 208.00±13.49 | 1.41±0.04 |
|  |  |  | |  |  |  |  |  |  |  |  |
| ***PGD1::OsDXR*** | **11** | N.D. | | 2.89±0.17 (1.5) | 112.58±10.24 (58.0) | 76.66±3.11 (39.5) | N.D. | 2.13±0.22 (1.1) | N.D. | 194.27±13.29 | 1.44±0.08 |
|  | **22** | N.D. | | 2.49±0.20 (1.6) | 81.13±6.49 (52.9) | 68.02±3.02 (44.4) | N.D. | 1.65±0.25 (1.1) | N.D. | 153.30±9.86 | 1.17±0.05 |
|  | **24** | N.D. | | 2.49±0.22 (1.3) | 108.95±9.47 (58.0) | 74.81±2.60 (39.8) | N.D. | 1.72±0.11 (0.9) | N.D. | 187.97±11.89 | 1.43±0.08 |
|  |  |  | |  |  |  |  |  |  |  |  |
| ***PGD1::OsDXS2*** | **Average** | N.D. | | 2.67±0.10 (1.3) | 115.07±8.12 (57.3) | 81.34±2.92 (40.5) | N.D. | 1.80±0.21 (0.9) | N.D. | 200.88±11.03 | 1.39±0.05 |
| ***PGD1::OsDXR*** | **Average** | N.D. | | 2.62±0.13 (1.5) | 100.89±9.93 (56.5) | 73.17±2.63 (41.0) | N.D. | 1.83±0.15 (1.0) | N.D. | 178.51±12.74 | 1.34±0.09 |
|  |  |  | |  |  |  |  |  |  |  |  |

All data are expressed as the mean values ± standard error from three independent experiments using mature seeds, the unit of carotenoid content is μg/g dry weight seeds, ‘%’ is the ratio of each carotenoid composition to the total carotenoid amount, and β/α is the ratio of β,β-carotenoids to β,Ɛ-carotenoids.

**Table S2.** Chlorophyll content in the leaves of transgenic rice plants

| **Plants** | **Line No.** | **Chlorophyll a** | | **Chlorophyll b** | **Total chlorophylls** |
| --- | --- | --- | --- | --- | --- |
|  |  |  | |  |  |
| **NT** | **IM** | 1.62+0.05 | | 0.39+0.01 | 2.01+0.06 |
|  |  |  | |  |  |
| ***PGD1::OsDXS2*** | **5** | 1.47+0.03 | | 0.34+0.01 | 1.81+0.03 |
|  | **32** | 1.36+0.02 | | 0.33+0.01 | 1.70+0.03 |
|  | **42** | 1.49+0.02 | | 0.34+0.01 | 1.83+0.03 |
|  |  |  | |  |  |
| ***PGD1::OsDXR*** | **11** | 1.46+0.02 | | 0.35+0.02 | 1.81+0.04 |
|  | **22** | 1.30+0.03 | | 0.30+0.01 | 1.60+0.04 |
|  | **24** | 1.41+0.03 | | 0.32+0.00 | 1.73+0.03 |
|  |  |  | |  |  |
| ***PGD1::OsDXS2*** | **Average** | 1.44+0.04 | | 0.34+0.00 | 1.78+0.04 |
| ***PGD1::OsDXR*** | **Average** | 1.39+0.05 | | 0.32+0.01 | 1.71+0.06 |
|  |  | |  |  |  |

All data are expressed as the mean values ± standard error from three independent experiments using the leaves of 10 days-old seedlings, the unit of chlorophyll content is μg/mg leaves.

**Table S3.** Carotenoid content and composition in mature seeds of transgenic rice plants

| **Plants** | **Line No.** | | **Carotenoid content (%)** | | | | | | | **Total carotenoid** | **β/α Ratio** |
| --- | --- | --- | --- | --- | --- | --- | --- | --- | --- | --- | --- |
|  |  |  | **Lycopene** | **α-Carotene** | **β-Carotene** | **Lutein** | **β-Cryptoxanthin** | **Zeaxanthin** | **Violaxanthin** |  |  |
|  | |  |  |  |  |  |  |  |  |  |  |
| **NT** | | **IM** | N.D. | N.D. | 0.04±0.01 (18.9) | 0.13±0.00 (65.6) | N.D. | 0.03±0.00 (15.4) | N.D. | 0.19±0.01 | 0.52±0.07 |
|  | |  |  |  |  |  |  |  |  |  |  |
| ***PGD1::OsDXS2*** | | **5** | N.D. | N.D. | 0.04±0.01 (18.0) | 0.14±0.00 (62.9) | N.D. | 0.04±0.00 (19.1) | N.D. | 0.23±0.01 | 0.59±0.05 |
|  | | **32** | N.D. | N.D. | 0.03±0.01 (16.7) | 0.12±0.00 (65.4) | N.D. | 0.03±0.00 (17.8) | N.D. | 0.18±0.00 | 0.53±0.02 |
|  | | **42** | N.D. | N.D. | 0.06±0.02 (17.8) | 0.21±0.01 (66.3) | N.D. | 0.05±0.00 (15.9) | N.D. | 0.31±0.01 | 0.51±0.01 |
|  | |  |  |  |  |  |  |  |  |  |  |
| ***PGD1::OsDXR*** | | **11** | N.D. | N.D. | 0.03±0.01 (13.7) | 0.13±0.01 (67.9) | N.D. | 0.04±0.00 (18.4) | N.D. | 0.19±0.01 | 0.47±0.03 |
|  | | **22** | N.D. | N.D. | 0.03±0.01 (16.9) | 0.10±0.00 (66.0) | N.D. | 0.03±0.00 (17.1) | N.D. | 0.15±0.00 | 0.51±0.05 |
|  | | **24** | N.D. | N.D. | 0.03±0.01 (16.0) | 0.12±0.01 (68.0) | N.D. | 0.03±0.00 (16.0) | N.D. | 0.17±0.01 | 0.47±0.03 |
|  | |  |  |  |  |  |  |  |  |  |  |
| ***Glb:stPAC*** | | **25** | 0.03±0.02 (0.8) | 0.44±0.03 (11.0) | 2.48±0.44 (61.7) | 0.56±0.03 (13.8) | 0.33±0.01 (8.1) | 0.19±0.03 (4.6) | N.D. | 4.03±0.52 | 2.97±0.35 |
|  | |  |  |  |  |  |  |  |  |  |  |
| ***PGD1::OsDXS2_Glb:stPAC*** | | **1** | 0.26±0.14 (1.2) | 5.75±0.22 (26.5) | 14.36±1.64 (66.2) | 0.38±0.02 (1.8) | 0.82±0.04 (3.8) | 0.08±0.02 (0.4) | 0.03±0.00 (0.2) | 21.67±2.04 | 3.26±0.22 |
|  | | **4** | 0.02±0.01 (0.2) | 1.85±0.14 (21.3) | 5.89±0.82 (68.0) | 0.35±0.01 (4.0) | 0.46±0.02 (5.3) | 0.08±0.01 (0.9) | 0.02±0.00 (0.3) | 8.66±0.97 | 3.23±0.23 |
|  | | **6** | 0.01±0.00 (0.1) | 2.29±0.19 (21.7) | 7.41±0.92 (70.3) | 0.30±0.02 (2.8) | 0.46±0.03 (4.4) | 0.05±0.01 (0.5) | 0.03±0.00 (0.2) | 10.55±1.10 | 3.39±0.16 |
|  | |  |  |  |  |  |  |  |  |  |  |
| ***PGD1::OsDXR_Glb:stPAC*** | | **3** | N.D. | 0.40±0.03 (13.0) | 1.83±0.12 (59.3) | 0.44±0.02 (14.3) | 0.34±0.02 (11.0) | 0.08±0.00 (2.4) | N.D. | 3.08±0.15 | 2.59±0.13 |
|  | | **16** | N.D. | 0.18±0.02 (14.1) | 0.67±0.03 (51.6) | 0.29±0.03 (22.3) | 0.09±0.01 (7.2) | 0.06±0.01 (4.9) | N.D. | 1.29±0.02 | 1.58±0.11 |
|  | | **17** | N.D. | 0.27±0.02 (16.4) | 0.79±0.08 (47.9) | 0.39±0.02 (23.9) | 0.11±0.01 (6.6) | 0.08±0.01 (5.1) | N.D. | 1.65±0.10 | 1.38±0.12 |
|  | |  |  |  |  |  |  |  |  |  |  |
|  | |  |  |  |  |  |  |  |  |  |  |
| ***PGD1::OsDXS2*** | | **Average** | N.D. | N.D. | 0.04±0.01 (17.6) | 0.16±0.03 (65.0) | N.D. | 0.04±0.01 (17.4) | N.D. | 0.24±0.04 | 0.54±0.02 |
| ***PGD1::OsDXR*** | | **Average** | N.D. | N.D. | 0.03±0.00 (15.4) | 0.12±0.01 (67.4) | N.D. | 0.03±0.00 (17.2) | N.D. | 0.17±0.01 | 0.49±0.01 |
| ***PGD1::OsDXS2_Glb:stPA*** | | **Average** | 0.10±0.08 (0.7) | 3.29±1.23 (24.2) | 9.22±2.61 (67.7) | 0.34±0.03 (2.5) | 0.58±0.12 (4.3) | 0.07±0.01 (0.5) | 0.03±0.00 (0.2) | 13.63±4.06 | 3.29±0.05 |
| ***PGD1::OsDXR_Glb:stPAC*** | | **Average** | N.D. | 0.28±0.06 (14.2) | 1.09±0.37 (54.6) | 0.37±0.05 (18.6) | 0.18±0.08 (9.0) | 0.07±0.01 (3.7) | N.D. | 2.01±0.55 | 1.85±0.37 |
|  | |  |  |  |  |  |  |  |  |  |  |

All data are expressed as the mean values ± standard error from three independent experiments using mature seeds, the unit of carotenoid content is μg/g dry weight seeds, ‘%’ is the ratio of each carotenoid composition to the total carotenoid amount, and β/α is the ratio of β,β-carotenoids to β,Ɛ-carotenoids.

| **Accession No.** | **Gene Names** |  | **NT^‡^** |  | ***PGD1::OsDXS2*** | | |  | ***PGD1::OsDXR*** | | |  |
| --- | --- | --- | --- | --- | --- | --- | --- | --- | --- | --- | --- | --- |
|  |  |  | Ilmi |  | 5 | 32 | 42 |  | 11 | 22 | 24 |  |
|  |  |  |  |  |  |  |  |  |  |  |  |  |
| Os05g33840 | ***OsDXS1*** |  | 0.039 ± 0.0014 |  | 3.070 ± 0.2597 | 3.072 ± 0.1444 | 3.959 ± 0.3086 |  | 6.669 ± 0.677 | 5.881 ± 0.6539 | 5.104 ± 0.6853 |  |
| Os07g09190 | ***OsDXS2*** |  | 0.004 ± 0.0003 |  | 238.6 ± 39.5391 | 484.0 ± 70.6334 | 596.2 ± 64.4097 |  | 4.023 ± 0.4554 | 2.013 ± 0.2096 | 3.031 ± 0.3940 |  |
| Os06g05100 | ***OsDXS3*** |  | 0.087 ± 0.0076 |  | 0.998 ± 0.1199 | 1.205 ± 0.0874 | 1.033 ± 0.1109 |  | 1.106 ± 0.1707 | 0.825 ± 0.0897 | 0.687 ± 0.0934 |  |
| Os01g01710 | ***OsDXR*** |  | 0.085 ± 0.0025 |  | 1.417 ± 0.1671 | 1.685 ± 0.1058 | 1.636 ± 0.1774 |  | 20.512 ± 2.0437 | 10.145 ± 1.0318 | 9.105 ± 1.2950 |  |
| Os03g52170 | ***OsIspH1*** |  | 2.000 ± 0.1570 |  | 0.735 ± 0.0928 | 1.051 ± 0.0982 | 0.792 ± 0.1153 |  | 1.249 ± 0.1257 | 1.177 ± 0.1184 | 1.213 ± 0.1611 |  |
| Os06g51290 | ***OsPSY1*** |  | 0.065 ± 0.0024 |  | 1.433 ± 0.1830 | 1.991 ± 0.1268 | 2.352 ± 0.3922 |  | 3.284 ± 0.3194 | 2.858 ± 0.3937 | 2.816 ± 0.3607 |  |
| Os12g43130 | ***OsPSY2*** |  | 0.045 ± 0.0005 |  | 3.893 ± 0.4569 | 4.396 ± 0.3136 | 5.660 ± 0.6010 |  | 10.578 ± 1.2132 | 8.710 ± 0.8921 | 10.358 ± 1.4696 |  |
| Os09g38320 | ***OsPSY3*** |  | 0.0003 ± 0.000 |  | 0.701 ± 0.1359 | 1.056 ± 0.2042 | 0.902 ± 0.2164 |  | 1.755 ± 0.1732 | 1.564 ± 0.2984 | 1.218 ± 0.1586 |  |
| Os09g38321 | ***OsPDS*** |  | 0.077 ± 0.0031 |  | 0.914 ± 0.1064 | 1.047 ± 0.1325 | 1.083 ± 0.1203 |  | 1.362 ± 0.1682 | 1.277 ± 0.1406 | 0.966 ± 0.1462 |  |
| Os09g38322 | ***OsLYCE*** |  | 0.014 ± 0.0010 |  | 1.435 ± 0.1816 | 3.127 ± 0.3967 | 3.391 ± 0.3998 |  | 1.966 ± 0.1958 | 1.578 ± 0.1704 | 3.165 ± 0.4434 |  |
| Os09g38323 | ***OsLYCB*** |  | 0.005 ± 0.0002 |  | 1.633 ± 0.2377 | 2.763 ± 0.2496 | 4.166 ± 0.4623 |  | 1.049 ± 0.1143 | 0.868 ± 0.1018 | 2.246 ± 0.3639 |  |
| Os02g57290 | ***OsCYP97A*** |  | 0.012 ± 0.0000 |  | 2.316 ± 0.2716 | 1.822 ± 0.1978 | 3.437 ± 0.3751 |  | 2.118 ± 0.2096 | 2.140 ± 0.2245 | 3.987 ± 0.5380 |  |
| Os02g07680 | ***OsCYP97B*** |  | 0.078 ± 0.0022 |  | 1.366 ± 0.1643 | 1.168 ± 0.0831 | 1.452 ± 0.1613 |  | 0.871 ± 0.0986 | 0.942 ± 0.1098 | 1.062 ± 0.1412 |  |
| Os10g39930 | ***OsCYP97C*** |  | 0.045 ± 0.0005 |  | 1.622 ± 0.1996 | 1.555 ± 0.1284 | 1.974 ± 0.2149 |  | 1.298 ± 0.1531 | 1.401 ± 0.1384 | 1.677 ± 0.2282 |  |
| Os03g03370 | ***OsBCH1*** |  | 0.045 ± 0.0026 |  | 1.759 ± 0.2110 | 1.984 ± 0.1314 | 2.364 ± 0.2888 |  | 3.560 ± 0.3680 | 2.878 ± 0.3055 | 2.403 ± 0.3060 |  |
| Os04g48880 | ***OsBCH2*** |  | 0.010 ± 0.0005 |  | 6.707 ± 0.8159 | 8.281 ± 0.5680 | 10.537 ± 1.1369 |  | 13.886 ± 1.3974 | 10.399 ± 3.0073 | 10.842 ± 2.2455 |  |
| Os10g38940 | ***OsBCH3*** |  | 0.069 ± 0.0043 |  | 1.397 ± 0.1660 | 1.918 ± 0.1710 | 2.311 ± 0.2576 |  | 3.988 ± 0.4580 | 3.022 ± 0.4949 | 2.745 ± 0.4293 |  |
| Os04g37619 | ***OsZEP1*** |  | 0.019 ± 0.0014 |  | 0.438 ± 0.0628 | 0.698 ± 0.0371 | 0.925 ± 0.0791 |  | 1.263 ± 0.1034 | 1.423 ± 0.0920 | 2.134 ± 0.1783 |  |
| Os04g31040 | ***OsVDE1*** |  | 0.092 ± 0.0037 |  | 2.281 ± 0.2950 | 1.987 ± 0.1887 | 2.844 ± 0.3565 |  | 1.902 ± 0.2078 | 1.709 ± 0.1765 | 2.096 ± 0.4114 |  |
| Os01g51860 | ***OsVDE2*** |  | 0.023 ± 0.0007 |  | 1.199 ± 0.1466 | 0.967 ± 0.0672 | 1.077 ± 0.1138 |  | 1.222 ± 0.1246 | 1.133 ± 0.1119 | 1.634 ± 0.2088 |  |
| Os01g03750 | ***OsNXS*** |  | 0.008 ± 0.0003 |  | 1.371 ± 0.1791 | 1.497 ± 0.1045 | 2.020 ± 0.2396 |  | 1.289 ± 0.1343 | 1.993 ± 0.1978 | 1.840 ± 0.2999 |  |
|  |  |  |  |  |  |  |  |  |  |  |  |  |

**Table S4.** Expression profiles of structure genes related to the biosynthesis of carotenoids in rice leaves.

All data are expressed as the mean values ± standard error from three technical replicates using mature seeds, and the expression level of the above genes are presented as the fold change values (2^-ΔΔCt^) relative to that of the NT plant (‡) which are calculated by the ΔCt equation against target genes and an *Ubi5* gene. The representative expression patterns of each genes are shown by the averaged values of them in three independent plants

**Table S5.** Expression profiles of structure genes related to the supplement of substrates into the biosynthesis of rice seed carotenes.

| **Plants** | **Line  No** | ***OsDXS1*** | | | ***OsDXS2*** | ***OsDXS3*** | ***OsDXR*** | ***OsIspH1*** | ***OsPSY1*** | ***OsPSY2*** | ***OsPSY3*** | ***OsPDS*** | ***OsLYCE*** | ***OsLYCB*** |
| --- | --- | --- | --- | --- | --- | --- | --- | --- | --- | --- | --- | --- | --- | --- |
|  |  |  | | |  |  |  |  |  |  |  |  |  |  |
| **NT^‡^** | **IM** | 0.017 ± 0.0007 | | | 0.013 ± 0.0027 | 0.056 ± 0.0090 | 0.033 ± 0.0016 | 0.053 ± 0.0021 | 0.020 ± 0.0004 | 0.029 ± 0.0011 | 0.014 ± 0.0007 | 0.165 ± 0.0143 | 0.001 ± 0.0001 | 0.008 ± 0.0001 |
|  |  |  | | |  |  |  |  |  |  |  |  |  |  |
| ***PGD1::OsDXS2*** | **5** | 0.775 ± 0.0404 | | | 4.202 ± 0.0493 | 0.594 ± 0.0537 | 0.682 ± 0.0516 | 0.741 ± 0.0450 | 1.271 ± 0.0489 | 1.147 ± 0.0453 | 1.245 ± 0.0458 | 0.909 ± 0.0492 | 0.463 ± 0.0653 | 0.719 ± 0.0632 |
|  | **32** | 1.210 ± 0.0868 | | | 5.339 ± 0.0694 | 1.491 ± 0.1605 | 1.067 ± 0.0890 | 1.062 ± 0.0860 | 1.624 ± 0.0752 | 1.575 ± 0.1266 | 1.883 ± 0.1003 | 1.228 ± 0.0806 | 0.956 ± 0.0762 | 0.854 ± 0.0905 |
|  | **42** | 0.770 ± 0.0345 | | | 4.138 ± 0.0331 | 1.160 ± 0.0611 | 0.651 ± 0.0498 | 0.799 ± 0.0429 | 1.207 ± 0.0504 | 1.213 ± 0.0461 | 1.208 ± 0.0404 | 0.834 ± 0.0391 | 0.420 ± 0.0534 | 0.675 ± 0.0548 |
|  |  |  | | |  |  |  |  |  |  |  |  |  |  |
| ***PGD1::OsDXR*** | **11** | 0.874 ± 0.0607 | | | 0.545 ± 0.0131 | 1.180 ± 0.1468 | 4.392 ± 0.3352 | 0.777 ± 0.0225 | 0.599 ± 0.0456 | 0.888 ± 0.0603 | 0.472 ± 0.0335 | 0.797 ± 0.0804 | 0.220 ± 0.0166 | 0.404 ± 0.0130 |
|  | **22** | 0.933 ± 0.0210 | | | 0.491 ± 0.0174 | 1.254 ± 0.0649 | 5.471 ± 0.2467 | 0.679 ± 0.0240 | 0.611 ± 0.0306 | 0.867 ± 0.0590 | 0.526 ± 0.0422 | 0.750 ± 0.0866 | 0.251 ± 0.0200 | 0.887 ± 0.1069 |
|  | **24** | 1.628 ± 0.0657 | | | 0.915 ± 0.0579 | 1.779 ± 0.1584 | 6.950 ± 0.3669 | 1.002 ± 0.0867 | 0.974 ± 0.0298 | 1.399 ± 0.0483 | 0.949 ± 0.0613 | 1.256 ± 0.1140 | 0.381 ± 0.0366 | 1.256 ± 0.0490 |
|  |  |  | | |  |  |  |  |  |  |  |  |  |  |
| ***Glb::stPAC*** | **25** | 0.818 ± 0.0552 | | | 0.676 ± 0.0079 | 1.105 ± 0.0977 | 0.773 ± 0.0815 | 0.765 ± 0.0579 | 1.073 ± 0.0511 | 1.301 ± 0.0640 | 1.144 ± 0.0510 | 0.945 ± 0.0951 | 0.365 ± 0.0989 | 0.545 ± 0.0575 |
|  |  |  | | |  |  |  |  |  |  |  |  |  |  |
| ***PGD1::OsDXS2***  ***_Glb::stPAC*** | **1** | 1.351 ± 0.0968 | | | 10.414 ± 0.1228 | 1.323 ± 0.0738 | 1.176 ± 0.0977 | 1.197 ± 0.1021 | 1.163 ± 0.0620 | 0.948 ± 0.0642 | 1.047 ± 0.0569 | 1.312 ± 0.0959 | 1.133 ± 0.3091 | 0.999 ± 0.1177 |
|  | **4** | 0.913 ± 0.0844 | | | 1.940 ± 0.0281 | 1.331 ± 0.1063 | 0.724 ± 0.0628 | 0.778 ± 0.0644 | 1.146 ± 0.0597 | 1.130 ± 0.0878 | 1.080 ± 0.0674 | 0.769 ± 0.0538 | 0.278 ± 0.0774 | 0.745 ± 0.0919 |
|  | **6** | 1.090 ± 0.0416 | | | 4.075 ± 0.0611 | 1.161 ± 0.0600 | 1.059 ± 0.0709 | 0.941 ± 0.0504 | 1.425 ± 0.0748 | 1.250 ± 0.0640 | 1.378 ± 0.0385 | 1.147 ± 0.0812 | 1.035 ± 0.1736 | 0.765 ± 0.1788 |
|  |  |  | | |  |  |  |  |  |  |  |  |  |  |
| ***PGD1::OsDXR***  ***_Glb::stPAC*** | **3** | 0.896 ± 0.0316 | | | 0.355 ± 0.0243 | 1.089 ± 0.1728 | 3.581 ± 0.7054 | 0.681 ± 0.0388 | 0.602 ± 0.0540 | 0.784 ± 0.0289 | 0.384 ± 0.0541 | 0.833 ± 0.0686 | 0.197 ± 0.0119 | 0.427 ± 0.0629 |
|  | **16** | 2.229 ± 0.1924 | | | 1.126 ± 0.0591 | 2.063 ± 0.1364 | 10.184 ± 0.6223 | 0.722 ± 0.0387 | 1.290 ± 0.0761 | 1.796 ± 0.1194 | 1.416 ± 0.0919 | 1.722 ± 0.1662 | 0.750 ± 0.0521 | 0.657 ± 0.2413 |
|  | **17** | 0.822 ± 0.0857 | | | 0.357 ± 0.0205 | 1.150 ± 0.0707 | 6.739 ± 0.6652 | 0.528 ± 0.0529 | 0.481 ± 0.0195 | 0.752 ± 0.0374 | 0.454 ± 0.0197 | 0.674 ± 0.0280 | 0.182 ± 0.0079 | 0.411 ± 0.0662 |
|  |  |  | | |  |  |  |  |  |  |  |  |  |  |
| ***PGD1::OsDXS2*** | **Average** | 0.918 ± 0.126 | | | 4.56 ± 0.338 | 1.082 ± 0.227 | 0.8 ± 0.116 | 0.867 ± 0.086 | 1.367 ± 0.112 | 1.312 ± 0.115 | 1.445 ± 0.19 | 0.99 ± 0.105 | 0.613 ± 0.149 | 0.749 ± 0.047 |
| ***PGD1::OsDXR*** | **Average** | 1.118 ± 0.127 | | | 5.476 ± 2.545 | 1.272 ± 0.055 | 0.986 ± 0.135 | 0.972 ± 0.122 | 1.245 ± 0.09 | 1.109 ± 0.088 | 1.168 ± 0.105 | 1.076 ± 0.161 | 0.815 ± 0.27 | 0.836 ± 0.082 |
| ***PGD1::OsDXS2***  ***_Glb::stPAC*** | **Average** | 1.145 ± 0.21 | | | 0.65 ± 0.115 | 1.404 ± 0.163 | 5.604 ± 0.642 | 0.819 ± 0.083 | 0.728 ± 0.107 | 1.051 ± 0.151 | 0.649 ± 0.131 | 0.934 ± 0.14 | 0.849 ± 0.214 | 0.284 ± 0.043 |
| ***PGD1::OsDXR***  ***_Glb::stPAC*** | **Average** | 1.316 ± 0.457 | | | 0.613 ± 0.257 | 1.434 ± 0.315 | 6.835 ± 1.907 | 0.644 ± 0.059 | 0.791 ± 0.252 | 1.111 ± 0.343 | 0.751 ± 0.333 | 1.076 ± 0.326 | 0.498 ± 0.079 | 0.376 ± 0.187 |
|  | | |  |  |  |  |  |  |  |  |  |  |  |  |

All data are expressed as the mean values ± standard error from three technical replicates using mature seeds, and the expression level of the above genes are presented as the fold change values (2^-ΔΔCt^) relative to that of the NT plant (‡) which are calculated by the ΔCt equation against target genes and an *Ubi5* gene. The representative expression patterns of each genes are shown by the averaged values of them in three independent plants

**Table S6.** Expression profiles of structure genes related to the xanthophyll biosynthesis in rice seeds.

| **Plants** | **Line  No** | ***OsCYP97A*** | ***OsCYP97B*** | ***OsCYP97C*** | ***OsBCH1*** | ***OsBCH2*** | ***OsBCH3*** | ***OsZEP1*** | ***OsVDE1*** | ***OsVDE2*** | ***OsNXS*** |
| --- | --- | --- | --- | --- | --- | --- | --- | --- | --- | --- | --- |
|  |  |  |  |  |  |  |  |  |  |  |  |
| **NT**^‡^ | **IM** | 0.001 ± 0.0003 | 0.022 ± 0.0026 | 0.004 ± 0.0007 | 0.121 ± 0.0022 | 0.025 ± 0.0015 | 0.045 ± 0.0032 | 0.001 ± 0.0001 | 0.017 ± 0.0008 | 0.040 ± 0.0008 | 0.025 ± 0.0002 |
|  |  |  |  |  |  |  |  |  |  |  |  |
| ***PGD1::OsDXS2*** | **5** | 0.933 ± 0.0451 | 1.338 ± 0.0585 | 0.835 ± 0.0727 | 0.656 ± 0.0700 | 0.724 ± 0.0638 | 0.723 ± 0.0615 | 1.390 ± 0.0313 | 0.752 ± 0.0440 | 0.764 ± 0.0497 | 1.045 ± 0.0676 |
|  | **32** | 1.420 ± 0.0707 | 1.434 ± 0.0822 | 0.843 ± 0.0895 | 0.802 ± 0.0820 | 1.028 ± 0.2139 | 1.110 ± 0.0974 | 1.212 ± 0.0296 | 1.206 ± 0.0783 | 1.001 ± 0.0746 | 1.152 ± 0.0746 |
|  | **42** | 1.283 ± 0.0886 | 1.153 ± 0.0630 | 0.908 ± 0.0666 | 0.356 ± 0.0256 | 0.599 ± 0.0455 | 0.712 ± 0.0371 | 1.604 ± 0.0386 | 0.814 ± 0.0627 | 0.740 ± 0.0384 | 1.203 ± 0.0567 |
|  |  |  |  |  |  |  |  |  |  |  |  |
| ***Glb::stPAC*** | **25** | 1.110 ± 0.1078 | 1.826 ± 0.1479 | 1.084 ± 0.1794 | 0.347 ± 0.0331 | 0.667 ± 0.0594 | 0.655 ± 0.0449 | 3.282 ± 0.0973 | 0.690 ± 0.0466 | 0.611 ± 0.0548 | 2.020 ± 0.1262 |
|  |  |  |  |  |  |  |  |  |  |  |  |
| ***PGD1::OsDXS2_Glb::stPAC*** | **1** | 1.470 ± 0.1074 | 1.371 ± 0.1135 | 1.150 ± 0.1975 | 0.882 ± 0.1130 | 1.106 ± 0.1053 | 1.175 ± 0.1065 | 1.281 ± 0.0539 | 1.291 ± 0.0893 | 1.048 ± 0.0886 | 1.215 ± 0.0782 |
|  | **4** | 1.472 ± 0.0799 | 1.178 ± 0.0692 | 0.768 ± 0.1175 | 0.331 ± 0.0385 | 0.658 ± 0.0546 | 0.858 ± 0.0745 | 1.597 ± 0.0581 | 0.890 ± 0.0803 | 0.663 ± 0.0596 | 1.379 ± 0.0796 |
|  | **6** | 0.789 ± 0.0495 | 1.158 ± 0.0364 | 1.063 ± 0.0807 | 0.745 ± 0.0682 | 1.028 ± 0.0701 | 0.695 ± 0.1091 | 1.674 ± 0.0369 | 1.049 ± 0.0562 | 0.985 ± 0.0551 | 1.026 ± 0.0356 |
|  |  |  |  |  |  |  |  |  |  |  |  |
| ***PGD1::OsDXS2*** | **Average** | 1.212 ± 0.126 | 1.308 ± 0.071 | 0.862 ± 0.02 | 0.605 ± 0.114 | 0.784 ± 0.11 | 0.848 ± 0.113 | 1.402 ± 0.098 | 0.924 ± 0.123 | 0.835 ± 0.072 | 1.133 ± 0.04 |
| ***PGD1::OsDXS2_Glb::stPAC*** | **Average** | 1.244 ± 0.227 | 1.236 ± 0.068 | 0.994 ± 0.116 | 0.653 ± 0.166 | 0.931 ± 0.138 | 0.909 ± 0.141 | 1.517 ± 0.12 | 1.077 ± 0.117 | 0.899 ± 0.119 | 1.207 ± 0.102 |
|  |  |  |  |  |  |  |  |  |  |  |  |

All data are expressed as the mean values ± standard error from three technical replicates using mature seeds, and the expression level of the above genes are presented as the fold change values (2^-ΔΔCt^) relative to that of the NT plant (‡) which are calculated by the ΔCt equation against target genes and an *Ubi5* gene. The representative expression patterns of each genes are shown by the averaged values of them in three independent plants

**Table S7.** The primer list used in vector construction and transgene analysis

| **Primers for vector construction** | | **Sequences (forward/reverse)** | **Product size (bp)** |
| --- | --- | --- | --- |
|  |  |  |  |
| *OsDXS2* | F1 | 5'-AAAAAGCAGGCTATGGCGCTCCAGGCATC-3' | 2200 |
|  | R1 | 5'-AGAAAGCTGGGTTCAGCTGAGCTGAAGTG-3' |  |
|  |  |  |  |
| *OsDXR* | F2 | 5'-AAAAAGCAGGCTATATGGCGCTCAAGGTCGT-3' | 1480 |
|  | R2 | 5'-AGAAAGCTGGGTCCTAGACAGGTACAGGGCT-3' |  |
|  |  |  |  |
| *attB* | GF | 5’-GGGGACAAGTTTGTACAAAAAAGCAGGCT-3’ |  |
|  | GR | 5’-GGGGACCACTTTGTACAAGAAAGCTGGGT- 3’ |  |
|  |  |  |  |
| *PGD1::Gene::PinII* | F3 | 5'-TGGTACCTAGATATGCCGAACAT-3' | 5127 / 4408 |
|  | R3 | 5'-GCGAATTCGTCGATTCATAGAAGATTAG-3' |  |
|  |  |  |  |
| **Primers for genomic DNA analysis** | | **Sequences (forward/reverse)** | **Product size (bp)** |
| **gDNA PCR** |  |  |  |
| *PGD1::OsDXS2* | F4 | 5'-CTCTTAACTTGCATGTCATAGTCTGATGTACTGTCC-3' | 2380 |
|  | R4 | 5'-GCTGAGCTGAAGTGCCTCCAATGGCCT-3' |  |
|  |  |  |  |
| *PGD1::OsDXR* | F4 | 5'-CTCTTAACTTGCATGTCATAGTCTGATGTACTGTCC-3' | 1597 |
|  | R5 | 5'-AGCATACTCCCTCGCCCACAGA-3' |  |
|  |  |  |  |
| *stPAC* | F6 | 5'-ATGAGCGTGGCTCTTCTCTGGG-3' | 2952 |
|  | R6 | 5'-TCAGATGAGGTCCTCCAGCATCAG-3' |  |
|  |  |  |  |
| **TaqMan PCR** |  |  |  |
| *Nos* | NF | 5'-GACGTTATTTATGAGATGGGTTTT-3' | 275 |
|  | NR | 5'-TGCGCGCTATATTTTGTTTTCTATCG-3' |  |
|  | NP | 5'-TAGAGTCCCGCAATTAT-3' |  |
|  |  |  |  |
| **Primers for qRT-PCR** | | **Sequences (forward/reverse)** | **Product size (bp)** |
|  |  |  |  |
| *OsDXS2* | F7 | 5'-CTCAAGTTGAGGTCCATGTTCTTGCCAG-3' | 275 |
|  | R7 | 5'-AGAAGATGGACAAGTCTAGGGTCACATTGCAGGGT-3' |  |
|  |  |  |  |
| *OsDXR* | F8 | 5'-ATGATCTGTGGGCGAGGGAGTATGCT-3' | 194 |
|  | R7 | 5'-AGAAGATGGACAAGTCTAGGGTCACATTGCAGGGT-3' |  |
|  |  |  |  |
| *stPAC* | F9 | 5'-TGACGCACAGGATGTTCA-3' | 144 |
|  | R9 | 5'-GGTAGAGGTTGGTGATGGT-3' |  |
|  |  |  |  |
| *Ubi5* | U5F | 5'-GAAGTAAGGAAGGAGGAGGA-3' | 100 |
|  | U5R | 5'-AAGGTGTTCAGTTCCAAGG-3' |  |

| **Gene** | **Accession No.** | **Primer sequences (forward/reverse)** | **Product size (bp)** |
| --- | --- | --- | --- |
|  |  |  |  |
| *OsDXS1* | Os05g33840 | 5'-GAGATCAGTGAATTGTATATTAGTCGGCG-3' | 110 |
|  |  | 5'-CGGGTCTCCGTCCACGAACAACTGAAGAGC-3' |  |
|  |  |  |  |
| *OsDXS2* | Os07g09190 | 5'-GGGGGAGGTTCCAGTAAGAA-3' | 119 |
|  |  | 5'-TCATTTTGCATTTGGAAGCA-3' |  |
|  |  |  |  |
| *OsDXS3* | Os06g05100 | 5'-GCTGTTTCATGGATATTCTTCAGTCTTTC-3' | 206 |
|  |  | 5'-CCTTCCTGTTACAAGCACTTGATAGGG-3' |  |
|  |  |  |  |
| *OsDXR* | Os01g01710 | 5'-GCTCCATGCATAGTCAGCAG-3' | 106 |
|  |  | 5'-GCACGGACGAACGATTTATT-3' |  |
|  |  |  |  |
| *OsIspH1* | Os03g52170 | 5'-CTGATGGCTTGGTGAAGGTT-3' | 101 |
|  |  | 5'-CAGCACATGCCGTAGTATGC-3' |  |
|  |  |  |  |
| *OsIspH2* | Os03g52180 | 5'-GCACAAGGAGACATGAGATGCTAGTATGG-3' | 124 |
|  |  | 5'-CCCAGATCACCAAATATGCACAAATAGTTC-3' |  |
|  |  |  |  |
| *OsPSY1* | Os06g51290 | 5'-GGGAAGATGATGAGCAGGTTA-3' | 120 |
|  |  | 5'-GCATTTTCCCTATACATGCT-3' |  |
|  |  |  |  |
| *OsPSY2* | Os12g43130 | 5'-TGTATGCCATAAGCCTGCCAC-3' | 122 |
|  |  | 5'-TATGCTTCTTGAACTGTGGGG-3' |  |
|  |  |  |  |
| *OsPSY3* | Os09g38320 | 5'-TGTAAGATGGGTATGTACCCC-3' | 124 |
|  |  | 5'-TGAGCTCATGCTAATGATCCT-3' |  |
|  |  |  |  |
| *OsPDS* | Os03g08570 | 5'-TGTGTCATCATCCCCTAGTCA-3' | 139 |
|  |  | 5'-TCAGCTCTCAGTCACAAATC-3' |  |
|  |  |  |  |
| *OsLYCE* | Os01g39960 | 5'-GTATGGCAGCGTTCACAGGGAC-3' | 64 |
|  |  | 5'-GCCAGCGTCATAGCATCGTCTC-3' |  |
|  |  |  |  |
| *OsLYCB* | Os02g09750 | 5'-CGTCCAGTACGACAAGCCGTA-3' | 189 |
|  |  | 5'-AAGGGCATGGCGTAGAGGAACG-3' |  |
|  |  |  |  |
| *OsCYP97A* | Os02g57290 | 5'-AAGTCTCAGCAACTCGAATCTGAGTTATG-3' | 155 |
|  |  | 5'-TGTAGCGCGAATCACCAAGATAAGCCTG-3' |  |
|  |  |  |  |
| *OsCYP97B* | Os02g07680 | 5'-ATCATCCACCAGTTGGCACCAAAAGCTGT-3' | 127 |
|  |  | 5'-CTTACCCTTGTATTGGTTGTTGTCTGC-3' |  |
|  |  |  |  |
| *OsCYP97C* | Os10g39930 | 5'-CAATGGCCTGTATATGAATGTAAGTCTG-3' | 140 |
|  |  | 5'-TCAACCTGTACGCCGATATTTACCACAT-3' |  |
|  |  |  |  |
| *OsBCH1* | Os03g03370 | 5'-TCGAGAACGTGCCCTACTTCC-3' | 119 |
|  |  | 5'-ACCCACCTCCTCCAACTCCTT-3' |  |
|  |  |  |  |
| *OsBCH2* | Os04g48880 | 5'-AGAGCTGGAGAAGGAGCTTGC-3' | 120 |
|  |  | 5'-AAAAATCTTGCAAGGCAAAGG-3' |  |
|  |  |  |  |
| *OsBCH3* | Os10g38940 | 5'-TCAAGAAGAGGATCAAGAGGA-3' | 129 |
|  |  | 5'-AGGAAAAGCCAAAACAAAAGCG-3' |  |
|  |  |  |  |
| *OsZEP1* | Os04g37619 | 5'-AGGCTGCGTCACCGGCGACCG-3' | 126 |
|  |  | 5'-CGGCTAATAACCCTTGTAACT-3' |  |
|  |  |  |  |
| *OsVDE1* | Os04g31040 | 5'-CTAAGGTAGCTTCTAGTTGCCACTCCAG-3' | 154 |
|  |  | 5'-ATTGTAATAATGGGGATGCATTAGCAG-3' |  |
|  |  |  |  |
| *OsVDE2* | Os01g51860 | 5'-ACAGGGTGATGTGTTCTAACGGCACAG-3' | 155 |
|  |  | 5'-ATATTTGTAGTCTAGTGGGTGCAACAGTG-3' |  |
|  |  |  |  |
| *OsNXS* | Os01g03750 | 5'-TCGACCTCTTCGCCGCAAGGCAG-3' | 195 |
|  |  | 5'-TACACCATCTAAGCAATATGAACCTACATACAATCG-3' |  |
|  |  |  |  |
| *OsUbi5* | Os01g22490 | 5'-GAAGTAAGGAAGGAGGAGGA-3' | 100 |
|  |  | 5'-AAGGTGTTCAGTTCCAAGG-3' |  |
|  |  |  |  |

**Table S8.** The primer list used in expression analysis of rice carotenogenic genes
